# Highly efficient nitrogen fixation over S-scheme heterojunction photocatalysts with enhanced active hydrogen supply

**DOI:** 10.1093/nsr/nwae093

**Published:** 2024-03-09

**Authors:** Tong Bao, Yamin Xi, Chaoqi Zhang, Peiyang Du, Yitong Xiang, Jiaxin Li, Ling Yuan, Chengzhong Yu, Chao Liu

**Affiliations:** School of Chemistry and Molecular Engineering, East China Normal University, Shanghai 200241, China; School of Chemistry and Molecular Engineering, East China Normal University, Shanghai 200241, China; School of Chemistry and Molecular Engineering, East China Normal University, Shanghai 200241, China; School of Chemistry and Molecular Engineering, East China Normal University, Shanghai 200241, China; School of Chemistry and Molecular Engineering, East China Normal University, Shanghai 200241, China; School of Chemistry and Molecular Engineering, East China Normal University, Shanghai 200241, China; School of Chemistry and Molecular Engineering, East China Normal University, Shanghai 200241, China; School of Chemistry and Molecular Engineering, East China Normal University, Shanghai 200241, China; Australian Institute for Bioengineering and Nanotechnology, The University of Queensland, Brisbane 4072, Australia; School of Chemistry and Molecular Engineering, East China Normal University, Shanghai 200241, China

**Keywords:** photocatalysis, nitrogen fixation, S-scheme heterojunction, active hydrogen, ammonia

## Abstract

Photocatalytic N_2_ fixation is a promising strategy for ammonia (NH_3_) synthesis; however, it suffers from relatively low ammonia yield due to the difficulty in the design of photocatalysts with both high charge transfer efficiency and desirable N_2_ adsorption/activation capability. Herein, an S-scheme CoS_x_/ZnS heterojunction with dual active sites is designed as an efficient N_2_ fixation photocatalyst. The CoS_x_/ZnS heterojunction exhibits a unique pocket-like nanostructure with small ZnS nanocrystals adhered on a single-hole CoS_x_ hollow dodecahedron. Within the heterojunction, the electronic interaction between ZnS and CoS_x_ creates electron-deficient Zn sites with enhanced N_2_ chemisorption and electron-sufficient Co sites with active hydrogen supply for N_2_ hydrogenation, cooperatively reducing the energy barrier for N_2_ activation. In combination with the promoted photogenerated electron-hole separation of the S-scheme heterojunction and facilitated mass transfer by the pocket-like nanostructure, an excellent N_2_ fixation performance with a high NH_3_ yield of 1175.37 μmol g^−1^ h^−1^ is achieved. This study provides new insights into the design of heterojunction photocatalysts for N_2_ fixation.

## Teaser

A pocket-like S-scheme CoS_x_/ZnS heterojunction with purpose-designed interfacial dual active sites has been synthesized for photocatalytic N_2_ fixation, achieving a high NH_3_ production rate of 1175.37 μmol g^−1^ h^−1^.

## INTRODUCTION

Ammonia (NH_3_) is one of the most important chemicals and has widespread applications in modern agriculture and industry [1–3]. The current industrial manufacture of NH_3_ relies on the famous Haber-Bosch process, using H_2_ and N_2_ as raw materials, which requires high temperatures and high pressure, consumes 1%–3% of global power supply and releases huge amounts of CO_2_ [4–6]. Photocatalytic N_2_ fixation offers a green and sustainable alternative approach for NH_3_ production under ambient conditions, using N_2_ and H_2_O as the source and sunlight as the energy input [7–9]. To date, various semiconducting photocatalysts, such as metal oxides [10], carbon nitride [11], metal sulfides [12], layered double hydroxides [13] and metal-organic frameworks (MOFs) [14], have been developed for N_2_ fixation. However, most single-component photocatalysts suffer from serious electron-hole recombination with restricted N_2_ fixation performance. To overcome this challenge, construction of heterojunction photocatalysts by combining two different semiconductors is one of the most promising strategies [15–17]. Recently, an S-scheme heterojunction composed of reduction and oxidation photocatalysts was proposed by Yu and co-workers [18]. The unique transfer path of photogenerated charge carriers enables the heterojunctions to possess efficient charge separation and strong redox ability [19–22]. To date, various inorganic, organic or inorganic-organic hybrid semiconducting materials have been used to construct S-scheme heterojunctions for different photocatalytic reactions such as hydrogen evolution [23,24], CO_2_ reduction [25,26], N_2_ fixation [27,28] and pollutant degradation [29,30]. However, most S-scheme heterojunction preparation focuses on the high charge separation efficiency; the design of the electronic structures of active sites within the heterojunction, specifically for N_2_ fixation, is largely overlooked.

The elaborate design of the photocatalysts at the molecular level, to reduce the energy barrier of the complex six-electron-coupled six-proton transfer process during N_2_ fixation, is of great importance [31–33]. One of the prerequisites is to modulate the electronic structure of active sites with enhanced N_2_ interaction in the first step [34]. To date, strategies such as introducing vacancies [35,36], dopants [37,38] and strains [39] into the photocatalysts have been developed to promote the adsorption of N_2_. Once the N_2_ molecule is adsorbed, the followed hydrogenation process is considered as the rate-determining step [40]. In electrocatalysis, recent advances have demonstrated that the generation of active hydrogen can reduce the energy barrier of N_2_ hydrogenation and thus facilitate NH_3_ production [41–45]. Nevertheless, such an active hydrogen-mediated N_2_ activation strategy has rarely been applied in photocatalytic N_2_ fixation. It is hypothesized that the construction of an S-scheme heterojunction together with purpose-designed active sites toward enhanced N_2_ adsorption and active hydrogen formation has the potential to significantly increase photocatalytic NH_3_ production performance.

**Scheme 1.**
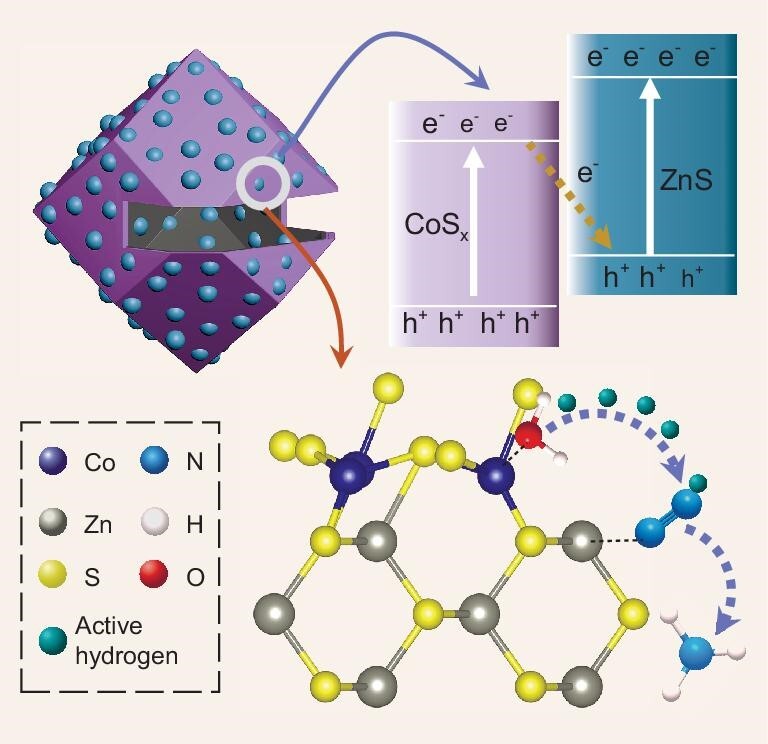
Schematic illustration of N_2_ fixation over a P-CoS_x_/ZnS S-scheme heterojunction.

Herein, we report an S-scheme CoS_x_/ZnS heterojunction photocatalyst for high-efficiency N_2_ fixation with rationally designed dual active sites at the interface toward enhanced N_2_ adsorption and active hydrogen supply (Scheme 1). The CoS_x_/ZnS heterojunction possesses a unique pocket-like morphology (thus denoted P-CoS_x_/ZnS), where small ZnS nanocrystals are anchored on an amorphous CoS_x_ hollow dodecahedron with a single rectangular hole. Experimental results combined with theoretical calculations have shown that the electron-deficient Zn sites and electron-sufficient Co sites within the heterojunction enhance the chemisorption of N_2_ molecules and facilitate active hydrogen generation for N_2_ hydrogenation, synergistically promoting N_2_ activation. Moreover, the charge separation is boosted by the S-scheme heterojunction, and the pocket-like nanostructure is beneficial for the mass transfer. Taken together, the P-CoS_x_/ZnS heterojunction shows excellent N_2_ fixation performance with a high NH_3_ yield of 1175.37 μmol g^−1^ h^−1^, superior to the single-component ZnS and CoS_x_, hollow CoS_x_/ZnS heterojunction with closed shell, and most reported photocatalysts.

## RESULTS AND DISCUSSION

### Characterization of P-CoS_x_/ZnS S-scheme heterojunction

**Figure 1.**
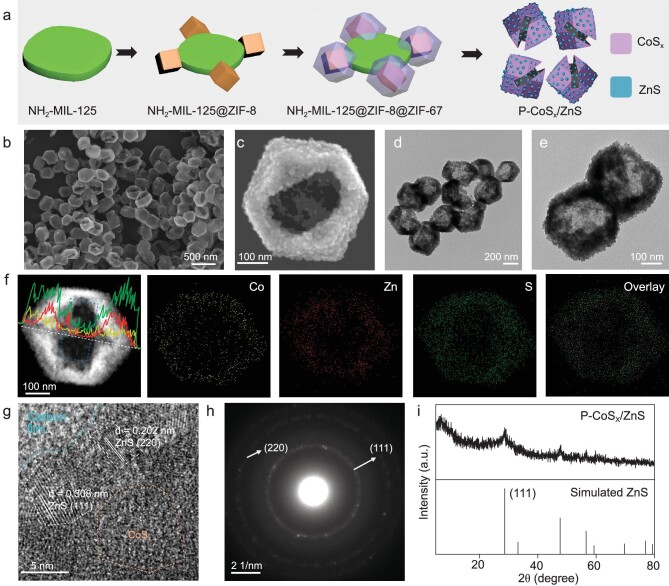
(a) Illustration of the synthesis process of P-CoS_x_/ZnS. (b and c) SEM images, (d and e) TEM images, (f) HAADF STEM image, line scanning spectra (inset) and corresponding element mapping images, (g) HRTEM image, (h) SEAD pattern, and (i) XRD pattern of P-CoS_x_/ZnS.

The synthetic process of P-CoS_x_/ZnS is schematically illustrated in Fig. 1a, using a ternary MOF heterostructure as a precursor followed by a sequential sulfidation-etching process. Ti-based MOFs (NH_2_-MIL-125, MIL = Materials of Institute Lavoisier) were synthesized via a solvothermal method as starting materials. Scanning electron microscopy (SEM) and transmission electron microscopy (TEM) images of NH_2_-MIL-125 (Fig. S1) show highly dispersed particles with a uniform cake-like morphology. The length and thickness were measured to be ∼630 and 140 nm, respectively. The X-ray diffraction (XRD) pattern shows the high crystallinity of NH_2_-MIL-125, in accordance with the simulated results (Fig. S2). By reacting NH_2_-MIL-125 with Zn(NO_3_)_2_ and 2-methylimidazole, ZIF-8 nanocrystals with an average diameter of ∼280 nm were grown on the four corners of NH_2_-MIL-125 (Fig. S3), forming the NH_2_-MIL-125@ZIF-8 composite with a tetrapod-like structure, according to a reported method [46]. In the XRD pattern, the diffraction peaks of both NH_2_-MIL-125 and zeolitic imidazolate framework (ZIF)-8 are detected (Fig. S4). By subsequently reacting NH_2_-MIL-125@ZIF-8 with Co(NO_3_)_2_ and 2-methylimidazole, ZIF-67 was selectively deposited onto ZIF-8 in NH_2_-MIL-125@ZIF-8, resulting in a ternary MOF heterostructure of NH_2_-MIL-125@ZIF-8@ZIF-67 following a reported protocol [47,48]. The tetrapod-like morphology is well preserved, with increased diameter and XRD peak intensities of ZIFs (Figs S5 and S6). The high-angle annular dark-field scanning TEM (HAADF STEM) and energy dispersive X-ray spectroscopy (EDX) mapping images of MIL-125@ZIF-8@ZIF-67 show that the Zn-rich core and Co-rich shell in the ZIF-8@ZIF-67 core-shell pods are attached on the corners of Ti-rich cake (Fig. S7a). Upon further combination with the line scanning spectra (Fig. S7b), the thickness of the ZIF-67 layer was estimated to be ∼40 nm.

The resultant NH_2_-MIL-125@ZIF-8@ZIF-67 hybrids were converted to P-CoS_x_/ZnS through a one-step sulfidation treatment. As shown in Fig. 1b and c, P-CoS_x_/ZnS exhibits a pocket-like morphology with a rectangular hole on a dodecahedral particle. At higher magnification (Fig. S8), both the external and interior surfaces are coarse with the adhesion of small nanoparticles. TEM images (Fig. 1d and e) directly reveal the hollow structure of P-CoS_x_/ZnS with a particle size, shell thickness and cavity diameter of ∼400, 40 and 320 nm, respectively. In addition, the width of the rectangular hole is determined to be ∼140 nm, well matched with the thickness of NH_2_-MIL-125 nanocake. The HAADF STEM and EDX element mapping images show the even distribution of Co, Zn and S elements in the skeleton of P-CoS_x_/ZnS (Fig. 1f). In the line scanning spectra (Fig. 1f, inset), the signal intensities of Co, Zn and S elements are weaker in the middle region (as marked by the blue line) due to the existence of a rectangular hole. The CoS_x_/ZnS molar ratio of P-CoSx/ZnS was determined to be ∼1/2 by inductive coupled plasma optical emission spectroscopy analysis.

From the high-resolution TEM (HRTEM) image (Fig. 1g), a crystalline/amorphous interface with clear boundaries was observed. The lattice fringes with planar distances of 0.308 and 0.202 nm correspond to the (111) and (220) planes of ZnS, respectively, consistent with the diffraction spots in the selected area electron diffraction (SAED) pattern (Fig. 1h). In the XRD pattern (Fig. 1i), only the diffraction peaks of ZnS are found, without the detection of crystalline cobalt sulfides. According to the literature [49,50] on sulfidation of ZIF-8 and ZIF-67 using a similar condition, with the preparation of P-CoS_x_/ZnS, the derived ZnS and CoS_x_ possessed a crystalline and amorphous nature respectively, in accordance with our results (Figs S9–S12). Taken together, it is inferred that P-CoS_x_/ZnS exhibits a distinctive pocket-like nanostructure, with ZnS nanocrystals adhered on the single-hole amorphous CoS_x_ hollow dodecahedron. Notably, even MOF-derived metal sulfides with hollow structures have been widely reported [51,52], most of them exhibiting a closed shell. A pocket-like hollow structure with a single hole on the surface is favorable for improving the diffusivity of guest species and making full use of the interior surface during catalytic reactions [53–55].

To investigate the formation process of P-CoS_x_/ZnS, the structural evolution of NH_2_-MIL-125@ZIF-8@ZIF-67 during sulfidation treatment was monitored at reaction times of 8 and 30 min. The resultant samples were denoted as NH_2_-MIL-125@ZIF-8@ZIF-67-S8 and S30, respectively. Via sulfidation for 8 min, the tetrapod-like morphology was well retained, with the detection of S element in the mapping images of MIL-125@ZIF-8@ZIF-67-S8 (Fig. S13, a1–a3). In addition, the line scanning spectra (Fig. S13, a4) show that the S signal is predominately distributed in the region of the Co-rich shell. In contrast, the signal intensity of S is weaker in the region of the Zn-rich core, indicating the preferential sulfidation of outer ZIF-67 into CoS_x_ shell. After 30 min, the nanocake body remains almost unchanged but the interior Zn-rich core is barely apparent, indicating the formation of hollow nanocages (Fig. S13, b1 and b2). Different from MIL-125@ZIF-8@ZIF-67-S8, the Co, S and Zn elements are evenly distributed in the hollow nanocages (Fig. S13, b3 and b4), suggesting that further sulfidation of ZIF-8 leads to the generation of ZnS nanocrystals that are deposited on the CoS_x_ shell, in accordance with the TEM/SEM observations in Fig. 1.

The changes in crystal and chemical structures were also studied by XRD and Fourier transform infrared (FTIR) spectroscopy (Figs S14 and S15). During the sulfidation process, the peaks of ZIFs are weakened at 8 min and then disappear at 30 min in the XRD patterns, corresponding to the sequential sulfidation of ZIF-67 and ZIF-8 in MIL-125@ZIF-8@ZIF-67. In contrast, the diffraction peaks of NH_2_-MIL-125 can still be observed due to the relatively high stability [50], in agreement with the TEM results. In the FTIR spectrum of MIL-125@ZIF-8@ZIF-67, the characteristic groups of NH_2_-MIL-125 (e.g. carboxyl group in 2-aminoterephthalic acid at 1250, 1385 and 1630 cm^−1^) and ZIFs (e.g. C−N band of 2-methylimidazole at 1146 cm^−1^) are observed [50,56]. Through sulfidation treatment, the peaks attributed to ZIFs were weakened and subsequently disappeared for MIL-125@ZIF-8@ZIF-67-S8 and S30, with the peaks of MIL-125 preserved, in agreement with the XRD results. By prolonging the reaction time to 3 h, the NH_2_-MIL-125 nanocake was further decomposed by breaking the coordination interaction between the Ti-O cluster and 2-aminoterephthalic acid linker [57], resulting in the formation of P-CoS_x_/ZnS. Collectively, the NH_2_-MIL-125@ZIF-8@ZIF-67 precursor experiences a sequential structural conversion process for the fabrication of P-CoS_x_/ZnS, which can be divided into three stages: (i) the selective sulfidation of the outer ZIF-67 layer into CoS_x_ shell; (ii) further sulfidation of ZIF-8 into ZnS nanocrystals deposited onto CoS_x_ with the generation of a hollow cavity; (iii) selective etching of NH_2_-MIL-125 for creating the single hole.

**Figure 2.**
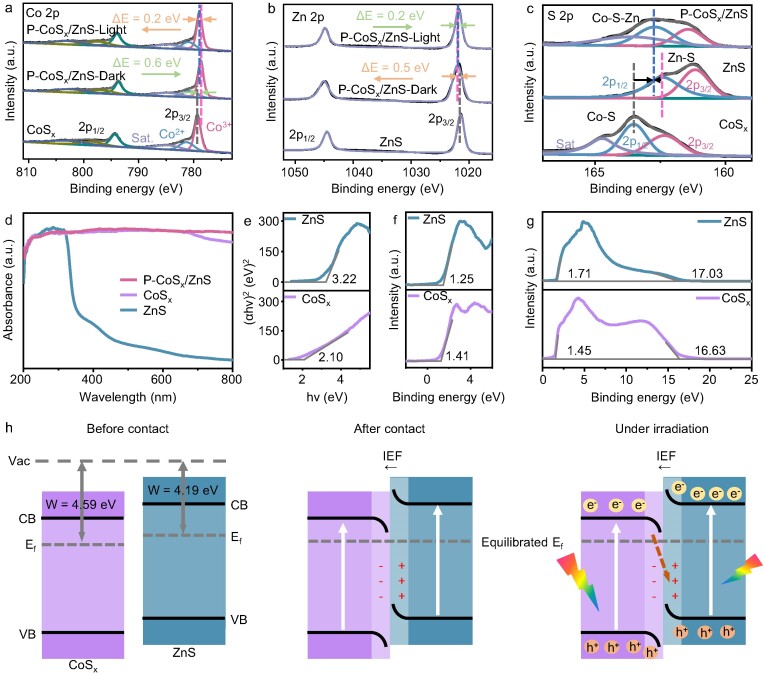
High-resolution XPS spectra of (a) Co 2p, (b) Zn 2p and (c) S 2p. (d) UV-vis DRS of different samples. (e) Tauc plots, (f) VB XPS spectra and (g) UPS spectra of CoS_x_ and ZnS. (h) Schematic illustration of S-scheme charge transfer mechanism between CoS_x_ and ZnS.

X-ray photoelectron spectroscopy (XPS) was used to investigate the surface chemical states and electronic structures of P-CoS_x_/ZnS. The XPS survey spectrum of P-CoS_x_/ZnS shows the coexistence of Zn, Co, O and S elements, while no signal of N element is observed (Fig. S16). Figure 2a displays the high-resolution Co 2p spectrum of CoS_x_, which can be divided into six peaks assigned to the 2p_3/2_ and 2p_1/2_ states of Co^3+^ at 779.3 and 794.3 eV, the 2p_3/2_ and 2p_1/2_ states of Co^2+^ at 781.3 and 798.5 eV, and two satellite peaks at 785.0 and 803.2 eV, respectively. Compared with CoS_x_, the binding energies of Co 2p peaks of P-CoS_x_/ZnS present a negative shift of ≈0.6 eV, indicative of the Co center with increased electron cloud density. For Zn 2p of ZnS (Fig. 2b), the two peaks at 1021.2 and 1044.5 eV are attributed to Zn 2p_3/2_ and Zn 2p_1/2_, respectively. In contrast to the negative shift of Co 2p, the binding energies of Zn 2p peaks in P-CoS_x_/ZnS are ≈0.5 eV more than ZnS, suggesting an electron-deficiency state of the Zn center.

The S 2p spectrum (Fig. 2c) of CoS_x_ can be fitted into two typical peaks of 2p_3/2_ and 2p_1/2_ orbitals of the Co−S bond at 162.4 and 163.5 eV, respectively, with one satellite peak at 164.7 eV. For ZnS, the two peaks at 161.2 and 162.4 eV were attributed to 2p_3/2_ and 2p_1/2_ orbitals of the Zn−S bond, respectively. For P-CoS_x_/ZnS, the peaks of 2p_3/2_ (161.4 eV) and 2p_1/2_ orbitals (162.7 eV) of metal-S bands are located between the Co−S bond of CoS_x_ and the Zn−S bond of ZnS, suggesting an interaction between CoS_x_ and ZnS by the Co−S−Zn bond. Based on the XPS results, it is also inferred that the electron transfer from ZnS to CoS_x_ may induce the formation of an internal electric field, providing potential driving force for photogenerated charge migration. Furthermore, when performing *in situ* irradiated XPS (ISIXPS) measurements under irradiation (Fig. 2a and b), the ISIXPS spectra of P-CoS_x_/ZnS show that the peaks of Co 2p positively shift and the peaks of Zn 2p negatively shift by 0.2 eV compared to those in the dark, indicating electron transfer from CoS_x_ to ZnS under illumination.

The optical property of P-CoS_x_/ZnS was investigated by Ultravioletvisible (UV-vis) diffuse reflectance spectra (DRS) in comparison with ZnS and CoS_x_. As shown in Fig. 2d, ZnS exhibits the absorption edge at ∼380 nm, corresponding to a bandgap of 3.22 eV calculated from the Tauc plots. In contrast, CoS_x_ with a dark black color shows intense light absorption in the whole UV-vis range owing to a narrowed bandgap of 2.10 eV [58]. Through combination of ZnS and CoS_x_, the light absorbance of P-CoS_x_/ZnS is slightly weaker than CoS_x_ but obviously stronger than ZnS. The valence band (VB) values of CoS_x_ and ZnS were determined as 1.62 and 1.46 eV by VB-XPS spectra (Fig. 2e and f). From the bandgap and VB values, the conduction band (CB) values of CoS_x_ and ZnS were calculated to be −0.48 and −1.76 eV, respectively. Thus, a matched and staggered band alignment between ZnS and CoS_x_ is illustrated in Fig. S17.

To directly probe the interfacial charge transfer within the P-CoS_x_/ZnS heterojunction, a Kelvin probe force microscopy (KPFM) measurement was carried out. An atomic force microscopy image of one P-CoS_x_/ZnS particle is shown in Fig. S18a. The lower contrast under illumination than in darkness of the KPFM images (Fig. S18b and c) implies the surface potential reduction under light irradiation, which was estimated to be 40.81 mV according to the potential-distance profiles (Fig. S18d). The KPFM observations suggest an electron accumulation of photogenerated electrons on ZnS in P-CoS_x_/ZnS, consistent with the ISIXPS results.

The Fermi level (E_F_) is important in determining the electron distribution in a heterojunction. Thus, the work function (Ф) of CoS_x_ and ZnS was measured by ultraviolet photoelectron spectrometry (UPS). The offset energies of CoS_x_ and ZnS were quantified as 16.63 and 17.03 eV (Fig. 2g), respectively. According to the formula Ф = hv-E_offset_ (hv = 21.22 eV), the Ф values of CoS_x_ and ZnS can be calculated as 4.59 and 4.19 eV (vs. vacuum level), respectively. Then, the E_F_ of CoS_x_ and ZnS is respectively determined to be −4.59 and −4.19 eV (vs. vacuum level) by Φ = E_V_ − E_F_, where the E_V_ is the potential of the vacuum as 0 eV.

Based on the results of XPS, ISIXPS, UV-vis DRS, KPFM and UPS, the charge transfer mechanism within the CoS_x_/ZnS heterojunction is depicted in Fig. 2h. As observed, the band position and E_F_ of ZnS are higher than CoS_x_. Upon contact between ZnS and CoS_x_, with the formation of an intimate interface, the free electrons in ZnS with higher E_F_ spontaneously migrate to CoS_x_, with lower E_F_ until an E_F_ level equilibrium is reached. The charge redistribution results in an interfacial built-in electric field with the direction pointing from ZnS to CoS_x_. Under irradiation, the electrons in ZnS and CoS_x_ are photoexcited from their VB to CB with holes left in the VB. The built-in electric field at the interface then drives the transfer of photogenerated electrons in the CB of CoS_x_ to consume the holes in the VB of ZnS, leading to the accumulation of electrons in the CB of ZnS and holes in the VB of CoS_x_. Such a charge transfer pathway follows an S-scheme mechanism, where both the stronger reduction ability of ZnS and oxidation ability of CoS_x_ can be preserved.

### Photocatalytic nitrogen fixation performance

**Figure 3.**
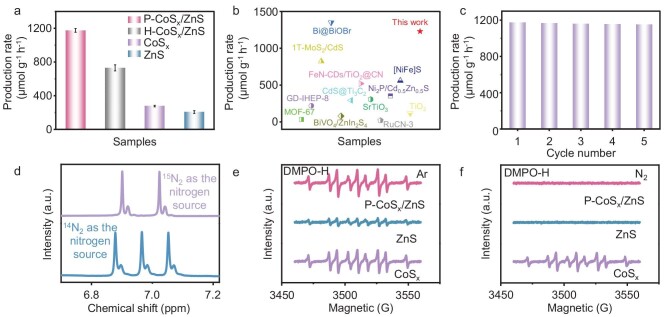
(a) NH_3_ production rate of P-CoS_x_/ZnS, H-CoS_x_/ZnS, CoS_x_ and ZnS. (b) Comparison of production rates of this work and reported photocatalysts. (c) Cycling test for NH_3_ production over P-CoS_x_/ZnS. (d) ^1^H NMR spectra of the reaction solution after photocatalytic N_2_ fixation using ^15^N_2_ and ^14^N_2_ as the feeding gas. ESR signals of DMPO-·H over P-CoS_x_/ZnS, ZnS and CoS_x_, under (e) Ar and (f) N_2_ atmosphere.

The photocatalytic NH_3_ production of P-CoS_x_/ZnS by N_2_ reduction was evaluated under simulated sunlight (AM 1.5 G) in N_2_-saturated pure water with CoS_x_ and ZnS for comparison. To demonstrate the advantages of the pocket-like nanostructure, CoS_x_/ZnS hollow nanocages with closed shells (H-CoS_x_/ZnS) were also fabricated by vulcanizing the core-shell ZIF-8@ZIF-67 hybrid (Figs S19–S22). Compared to P-CoS_x_/ZnS, H-CoS_x_/ZnS possesses almost the same optical property and electronic structures, and slight reduction in specific surface area and total pore volumes (Figs S23 and S24; Table S1). The NH_3_ concentration was quantified by ^1^H nuclear magnetic resonance (NMR) spectroscopy (Figs S25 and S26). As shown in Fig. 3a, ZnS and CoS_x_ exhibit an NH_3_ production rate of 205.98 and 276.67 μmol g^−1^ h^−1^ in 6 h, respectively. In contrast, the NH_3_ generation rate of P-CoS_x_/ZnS increases to 1175.37 μmol g^−1^ h^−1^, also higher than that for H-CoS_x_/ZnS (730.65 μmol g^−1^ h^−1^), indicating a positive contribution of both heterojunction and pocket-like morphology for improving N_2_ fixation performance. Such a high NH_3_ yield is one of the best of the reported photocatalysts to date (Fig. 3b). In addition, possible byproducts, including NO_3_^−^, N_2_H_4_ and H_2_, were barely detected during the photocatalytic process (Figs S27–S29), indicating a high selectivity of N_2_ reduction to NH_3_ over P-CoS_x_/ZnS.

To further evaluate the activity of photocatalytic NH_3_ production, the apparent quantum efficiencies (AQEs) were explored under monochromatic light irradiation with different wavelengths. As shown in Fig. S30, the AQE values are 7.56%, 3.85%, 1.78%, 0.92% and 0.55% at 365, 400, 450, 500 and 550 nm, respectively. Notably, the peak value of 7.56% at 365 nm is superior to most reported photocatalysts (Table S2). As a product of coupled water oxidation reaction, the production rate of O_2_ was determined to be 812.23 μmol g^−1^ h^−1^ for P-CoS_x_/ZnS, also higher than other samples (Fig. S31).

To verify the source of nitrogen in the produced NH_3_, ^14^N_2_/^15^N_2_ isotope labeling ^1^H NMR was conducted (Fig. 3d). When using high-purity ^15^N_2_ as the feeding gas, the ^1^H NMR spectrum shows typical double peaks of ^15^NH_4_^+^ products at chemical shifts of 6.90 and 7.02 ppm. For the ^1^H NMR spectrum obtained by using^14^N_2_, triple peaks of ^14^NH_4_^+^ are detected. Additionally, no NH_3_ was detected in the reaction system when changing the feeding gas to Ar (Fig. S32). The above results demonstrate that the produced NH_3_ originates from N_2_ fixation, rather than other possible nitrogenous sources.

In addition to activity, the photocatalytic stability of P-CoS_x_/ZnS was also assessed via cycling test. As shown in Fig. 3c, a negligible decrease in NH_3_ production was observed after five cycles, compared to the first cycle. In addition, the TEM image, XRD pattern and XPS spectra of used P-CoS_x_/ZnS (Figs S33 and S34) show well-maintained pocket-like morphology, crystalline structure and local coordination environment, indicating good photocatalytic stability.

To explore the photocatalytic mechanism of N_2_ fixation to NH_3_ over P-CoS_x_/ZnS, an *in situ* diffuse reflectance infrared Fourier transformation (DRIFT) spectroscopy measurement was performed (Fig. S35). The DRIFT spectra in the dark show two vibrational bands at 3426 and 1643 cm^−1^, assigned to the absorbed −OH group of H_2_O and absorbed N_2_, respectively. Under light irradiation for 10 min, the peaks of adsorbed H_2_O and N_2_ are weakened, with a new peak assigned to absorbed NH_3_ emerging at 1553 cm^−1^. As the reaction proceeds, the peak of NH_3_ gradually intensifies with the peak intensity of H_2_O, and N_2_ further reduces. The results of *in situ* DRIFT spectra suggest that the photocatalytic process over P-CoS_x_/ZnS experiences the activation of N_2_ in the presence of H_2_O to produce NH_3_.

Moreover, an electron spin resonance (ESR) measurement was performed to probe the generation of hydrogen radicals during the reaction process, where 5,5-dimethyl-pyrroline N-oxide (DMPO) was used as the spin-trapping agent. In the ESR spectra, nine strong peaks with a density ratio of approximately 1 : 1 : 2 : 1 : 2 : 1 : 2 : 1 : 1 were detected, which correspond to the spin product DMPO-H, verifying the generation of active hydrogen from water decomposition [41,42]. In the absence of N_2_, the DMPO-H signal intensities of ZnS_x_ are extremely weak, while those for CoS_x_ are much stronger. After integrating these two components, the signal intensities of P-CoS_x_/ZnS are further strengthened, indicating the promoted formation of active hydrogen by P-CoS_x_/ZnS (Fig. 3e). In the presence of N_2_, the DMPO-H signals of CoS_x_ were slightly weakened (Fig. 3f), while those for ZnS and P-CoS_x_/ZnS were almost undetectable. These observations suggest that the CoS_x_ component in P-CoS_x_/ZnS is mainly responsible for the generation of active hydrogen, which is rapidly consumed for the hydrogenation process of N_2_ fixation [43,45].

**Figure 4.**
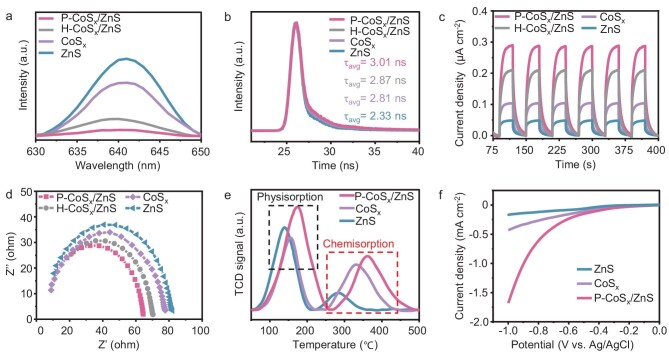
(a) PL spectra, (b) TRPL, (c) transient photocurrent response, (d) Nyquist plots, (e) N_2_-TPD spectra and (f) LSV curves of different samples.

The charge separation efficiencies of the four samples were investigated by photoluminescence (PL) spectra, time-resolved PL spectra (TRPL), photocurrent measurement and electrochemical impedance spectroscopy (EIS). The PL spectra in Fig. 4a show the lowest emission peak intensity of P-CoS_x_/ZnS among all samples, revealing the lowest recombination efficiency of photogenerated electron-hole pairs. The average PL lifetime (τ_avg_) of P-CoS_x_/ZnS was calculated to be 3.01 ns, which was longer than that of H-CoS_x_/ZnS (2.87 ns), CoS_x_ (2.81 ns) and ZnS (2.33 ns), indicating the longer lifetime of electron-hole pairs in P-CoS_x_/ZnS (Fig. 4b). In the photocurrent profile (Fig. 4c), the photocurrent density follows the order of P-CoS_x_/ZnS > H-CoS_x_/ZnS > CoS_x_ > ZnS. Figure 4d displays the EIS Nyquist plots, showing the lowest semicircle of P-CoS_x_/ZnS with the lowest charge transfer resistance. These observations are consistent with the NH_3_ production results, further suggesting the vital role of S-scheme heterojunctions and pocket-like structures in promoting charge separation and N_2_ fixation.

Subsequently, nitrogen temperature programmed desorption (N_2_-TPD) measurements were performed to investigate the adsorption of N_2_ over different samples. In the N_2_-TPD curves (Fig. 4e), two major adsorption peaks attributed to physisorption (130–180°C) and chemisorption (280–370°C) are observed. P-CoS_x_/ZnS exhibits higher chemisorption temperature (361°C) than CoS_x_ (331°C) and ZnS (284°C), indicating enhanced N_2_ chemisorption on P-CoS_x_/ZnS. Furthermore, linear sweep voltammetry (LSV) curves were acquired to explore N_2_ activation capability. As shown in Fig. S36, the current density of P-CoS_x_/ZnS in the presence of both N_2_ and light irradiation is stronger than that with only N_2_ or light irradiation, implying the occurrence of a photocatalytic N_2_ reduction reaction. Moreover, P-CoS_x_/ZnS also delivers the lowest onset potential and highest current density among all samples (Fig. 4f and Table S3), indicating higher N_2_ activation activity.

### DFT calculations

**Figure 5.**
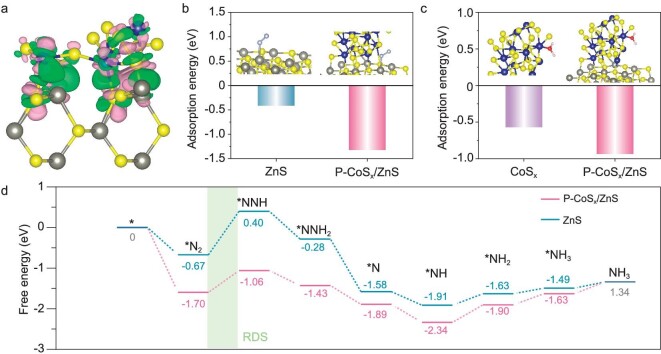
(a) Calculated charge difference distribution of P-CoS_x_/ZnS. (b) Calculated nitrogen molecule adsorption energies at the Zn sites of ZnS and P-CoS_x_/ZnS. (c) Calculated water molecule adsorption energies at the Co sites of CoS_x_ and P-CoS_x_/ZnS. (d) Free energy diagrams for photocatalytic nitrogen reduction to ammonia on ZnS and P-CoS_x_/ZnS. Color code: Co, blue; Zn, gray; S, yellow; O, red; H, white; N, light gray.

To further elucidate the origin of reinforced N_2_ fixation over P-CoS_x_/ZnS, density functional theory (DFT) calculations were performed. First, the spatial charge distribution at the CoS_x_/ZnS interface was explored by calculating the differential charge density (Fig. 5a), where the green and pink areas represent charge accumulation and depletion, respectively. The electrons are mainly accumulated around Co atoms but depleted from Zn atoms, indicating electron transfer from ZnS to CoS_x_ within the heterojunction. Such an interfacial electron transfer results in not only a built-in electric field pointing from ZnS to CoS_x_ without photoexcitation, but also electron-deficient Zn sites and electron-sufficient Co sites, in agreement with the XPS and UPS observations.

The experimental and theoretical calculation results collectively suggest that the ZnS and CoS_x_ in the P-CoS_x_/ZnS heterojunction serve as the active components for N_2_ adsorption/activation and H_2_O decomposition for active hydrogen generation, respectively. Therefore, the N_2_ and H_2_O adsorption energies of P-CoS_x_/ZnS were calculated and compared with single ZnS and CoS_x_. As shown in Fig. 5b, the adsorption energy of N_2_ for P-CoS_x_/ZnS was calculated as –1.32 eV, more negative than that for ZnS (–0.41 eV). Thus, N_2_ adsorption is thermodynamically more favorable on the electron-deficient Zn sites in P-CoS_x_/ZnS, in agreement with the N_2_-TPD results. Additionally, the more negative adsorption energy of H_2_O on P-CoS_x_/ZnS (–0.94 eV) than CoS_x_ (–0.57 eV) indicates boosted H_2_O adsorption on electron-sufficient Co sites in P-CoS_x_/ZnS. It is reported that strong adsorption of H_2_O can facilitate its splitting into active hydrogen [41,43,45], which is in accordance with the active hydrogen signals of P-CoS_x_/ZnS being stronger than those for CoS_x_ in the ESR spectra.

To aid in the understanding of the complex six-electron-coupled six-proton transfer process of N_2_ fixation, a free energy diagram based on the Gibbs free energy change (ΔG), as well as the corresponding adsorption configurations, are depicted in Fig. 5d and Figs S37 and 38. Generally, the N_2_ fixation pathway involves two main possible mechanisms, the distal and alternating mechanisms (Fig. S39) [40,59]. Considering that no N_2_H_4_ was detected in the reaction [60], we thereby focused on the distal pathway. As presented in Fig. 5d, the first hydrogenation process (*N_2_ → *NNH) was recognized as the rate-determining step (RDS) during the overall N_2_ fixation process for both ZnS and P-CoS_x_/ZnS. The ΔG of the RDS for P-CoS_x_/ZnS was calculated to be 0.64 eV, much lower than that for ZnS (1.07 eV), indicating that the heterojunction can largely reduce the energy barrier for N_2_ hydrogenation.

The experiment and DFT calculation results have clearly illustrated the important role of S-scheme heterojunctions with pocket-like morphology in enhancing N_2_ production performance as follows: (i) the construction of a S-scheme heterojunction promotes charge separation and enhances redox properties compared to single CoS_x_ and ZnS; (ii) the construction of electron-deficient Zn sites in ZnS can enhance the chemisorption of N_2_ molecules; (iii) the stronger adsorption of H_2_O on electron-sufficient Co sites in CoS_x_ is conducive to water decomposition toward active hydrogen generation, promoting the hydrogenation of N_2_ with a reduced energy barrier; (iv) the pocket-like morphology may facilitate the diffusion of N_2_ and desorption of NH_3_.

## CONCLUSION

In summary, an S-scheme P-CoS_x_/ZnS heterojunction with a unique pocket-like nanostructure has been synthesized for N_2_ fixation, with a high NH_3_ production rate of 1175.37 μmol g^−1^ h^−1^. In our design, the electron-deficient Zn sites are responsible for strengthening the chemisorption of N_2_ molecules, and the electron-sufficient Co sites promote the generation of active hydrogen for N_2_ hydrogenation with a reduced energy barrier. Contributed to even further by the S-scheme heterojunction with efficient photogenerated carrier separation and pocket-like nanostructure with enhanced mass transfer, excellent photocatalytic N_2_ fixation performance is eventually achieved. This work paves the way for photocatalyst design toward high-performance N_2_ fixation.

## METHODS

Details about the sample synthesis and characterization are included in the online supplementary data.

## Supplementary Material

nwae093_Supplemental_File
