# Supplementary material for: Highly efficient nitrogen fixation over S-scheme heterojunction photocatalysts with enhanced active hydrogen supply
Source: Natl Sci Rev. 2024 Mar 9;11(5):nwae093. doi: 10.1093/nsr/nwae093 (PMC10989659; doi:10.1093/nsr/nwae093)
Supplement: nwae093_Supplemental_File [file nwae093_supplemental_file.pdf]

Supplementary Data

Highly Efficient Nitrogen Fixation over S-Scheme Heterojunction Photocatalysts with Enhanced Active Hydrogen Supply

Tong Bao[1], Yamin Xi[1], Chaoqi Zhang[1], Peiyang Du[1], Yitong Xiang[1], Jiaxin Li[1], Ling Yuan[1], Chengzhong Yu[1, 2*], Chao Liu[1*]

[1]School of Chemistry and Molecular Engineering, East China Normal University, Shanghai 200241, China

[2]Australian Institute for Bioengineering and Nanotechnology, The University of Queensland, Brisbane, Queens-land 4072, Australia

*Corresponding author. Emails: czyu@chem.ecnu.edu.cn; c.yu@uq.edu.au; cliu@chem.ecnu.edu.cn

Materials and Synthetic procedures

Materials

Titanium isopropoxide (TPOT, 97%, Aldrich), 2-aminoterephthalate (99%, Aldrich), zinc nitrate hexahydrate ($Zn(NO_3)_2 \cdot 6H_2O$), cobalt nitrate hexahydrate ($Co(NO_3)_2 \cdot 6H_2O$) from Sinopharm Chemical Regent Co., Ltd, 2-methylimidazole (2-MeIM, Aldrich), N,N-dimethylformamide (DMF, 99%, Greagent), thioacetamide (TAA, 99%, Adamas-beta), cetyltrimethylammonium bromide (CTAB, Adamas-beta), methanol (AR, 99%, Adamas-beta), ethanol (AR, 99.7%, Adamas-beta), p-dimethylaminobenzaldehyde (AR, 99%, Adamas-beta), hydrochloric acid (HCl, AR, Shanghai Hushi Co., Ltd.), Deuterium Oxide (D_2O, 99%, Adamas-beta) and maleic acid (AR, 95%, Shanghai Shaoyuan Co., Ltd.) were used as received. Millipore water was used in all experiments.

Synthesis of NH_2-MIL-125

Cake-like NH_2-MIL-125 nanoparticles were synthesized according to a reported protocol [1]. Typically, 0.56 g of 2-aminoterephthalate was dissolved in 36 mL of DMF and 4 mL of methanol, followed by adding 0.6 mL of TPOT with stirring for 5 min. Then, the suspension was transferred into a 100 mL Teflon-line autoclave and heated at 150 ℃ for 24 h. The final products were collected by centrifugation and washed by DMF and methanol for three times. The obtained NH_2-MIL-125 was re-dispersed into 30 mL of methanol to make a stock solution for further use.

Synthesis of NH_2-MIL-125@ZIF-8

To synthesize NH_2-MIL-125@ZIF-8, 0.06 mL of NH_2-MIL-125 methanol solution, 3 mL of 25 mM 2-MeIM solution and 5mL of 25 mM $Zn(NO_3)_2 \cdot 6H_2O$ solution were allowed to react at room temperature for 4 h. The products were then collected by centrifugation, washed with methanol three times, and dispersed in 2 mL of methanol for further use.

Synthesis of NH_2-MIL-125@ZIF-8@ZIF-67

In detail, 1.65 mL of 80 mM 2-MeIM and 1 mL of 25 mM $Co(NO_3)_2 \cdot 6H_2O$ methanol solution was mixed with 2 mL of NH_2-MIL-125@ZIF-8 methanol solution. After reaction for 2 h at room temperature, the products were centrifugated, washed with methanol for three times and dried overnight.

Synthesis of P-CoS_x/ZnS

Typically, 25 mg of the resultant NH_2-MIL-125@ZIF-8@ZIF-67 was dispersed in 10 mL of ethanol. 10 mL of ethanol solution containing 0.0375 g TAA was then poured into the above

suspension by stirring for 5 min. Subsequently, the mixture was transferred into a 50 mL Teflon-line autoclave and kept at 180 ℃ for 3 h. After centrifugation, washing with ethanol for three times and drying overnight, the final products were obtained, denoted P-CoS_x/ZnS.

Synthesis of ZIF-8 and ZnS

To prepare ZIF-8, 0.712 g of $Zn(NO_3)_2 \cdot 6H_2O$ was dissolved into 30 mL of deionized water followed by adding 5 mL of 0.01M CTAB aqueous solution. 170 mL of deionized water containing 10.8 g of 2-MeIM was poured into the above mixture. After stirring for 3 h at room temperature, the products were collected, washed with methanol for four times and dried overnight. ZnS was synthesized by sulfidation of ZIF-8 using the same procedure with P-CoS_x/ZnS.

Synthesis of ZIF-67 and derived CoS_x

Typically, 50 mL of 0.16 M 2-MeIM solution and 50 mL of 0.04 M $Co(NO_3)_2$ $6H_2O$ solution were mixed with stirring for 2 min. By further aging at room temperature for 24 h, the products were collected by centrifugation, washed with methanol three times, and dried overnight. For the synthesis of CoS_x, ZIF-67 was vulcanized using the same procedure with P-CoS_x/ZnS.

Synthesis of H-CoS_x/ZnS

To obtain ZIF-8@ZIF-67, 50 mg of as-prepared ZIF-8 was dispersed in 10 mL of methanol. By further adding 4 mL of 0.2 M $Co(NO_3)_2$ $6H_2O$ methanol solution and 4 mL of 0.8 M 2-MeIM methanol solution, the mixture was stirred at room temperature for 24 h. The products were then collected by centrifugation, washed with methanol three times, and dried overnight. Through the same sulfidation treatment with P-CoS_x/ZnS, H-CoS_x/ZnS was fabricated.

Material Characterization

SEM images were collected by scanning electron microscope (HITACHI-S4800). TEM images were acquired with JEOL 2100F at 200 KV. The element analysis was conducted on a field-emission scanning electron microscope (FIB, Helios G4 UX, FEI Inc. USA) equipped with an X-ray energy dispersive spectrometer (EDS: X-Max 150T, Oxford, UK). XRD patterns were recorded using a Bruker D8 advanced X-ray diffractometer with Cu Kα radiation (λ = 0.154 nm). Fourier Transform Infrared Spectroscopy (FTIR) spectra were obtained via a Thermal-Nicolet iS50 spectrometer between 4000 and 500 cm^{-1}. The XPS and VB XPS spectra were obtained by using a PHI Quantera II ESCA System with Al Kα radiation at 1486.8 V. The $E_{VB\text{-}XPS}$ value

relative to standard hydrogen electrode (vs NHE) can be calculated according to formula: $E_{VB\text{-}NHE} = \varphi + E_{VB\text{-}XPS} - 4.44$, Where φ is the work function of the instrument is 4.65 eV [2,3]. In-situ XPS was conducted under the same condition, except that simulated sunlight (AM 1.5G) irradiation was introduced. The photo-irradiated Kelvin probe force microscopy (KPFM) (Bruker Dimension ICON) was used to test the surface potential of the samples. Ultraviolet photoelectron spectroscopy (UPS) studies were conducted by using an ESCALAB 250 XI analyzer with He (21.22 eV) as monochromatic light source. N_2 sorption isotherms were measured by using Micromeritics ASAP-2460 at −196 °C. The AQE measurement was performed by using monochromatic lights with different wavelengths. The AQE values were measured according to following formula: AQE = 100% × (number of molecules of ammonia generated × 3) / number of incident photons [4] . ^1H nuclear magnetic resonance (NMR) spectra were recorded on a Bruker AC-600FT spectrometer (600 MHz). In situ diffuse reflectance Fourier transform infrared spectroscopy experiment was operated in a reaction chamber (Harrick, Praying Mantis) which attached to an FTIR spectrometer (Thermal-Nicolet iS50 spectrometer) under a N_2 flow with H_2O steam. Dark adsorption was continued for 60 min before illumination. Electron spin resonance (ESR) spectra were recorded on a Bruker A300 ESR spectrometer. PL spectra were recorded on an Edinburgh FS5 spectrofluorometer in the range of 390 - 800 nm. The samples were dispersed in ethanol at the same concentration. Time-resolved photoluminescence (TRPL) spectra were collected on a FSL980 transient fluorescence spectrometer. Nitrogen temperature programmed desorption (N_2-TPD) was acquired on Autosorb-iQ-C chemisorption analyzer (Quantachrome, USA).

Photocatalytic N_2 fixation

10 mg of photocatalyst was dispersed in 12 mL of DI water by ultrasonication. After continuously bubbled with N_2 for 1 h, the photocatalytic reaction was initiated by the irradiation of simulated sunlight (AM 1.5G) using a 300 W xenon lamp as light source. 500 μL of the suspension was collected after 6 h and filtrated with 0.22 μm filters to remove the photocatalysts. Then, 250 μL of the filtered solution was diluted into 250 μL deionized water. The concentration of ammonia product was measured by NMR. 5 mL of the filtered and diluted reaction solution was mixed with 5 mL of 0.1 M HCl. By further adding 50 μL of D_2O and 5 mL of maleic acid (50 ppm), the mixture solution was diluted into the detection range for NMR test. The stand curve for NH_3

quantification was illustrated in Figure S25.

Detection of NO_3^-

A certain amount of reaction solution was taken out and diluted to 5 mL to the detection range. Then, 0.1 mL 1 M HCl and 0.01 mL 0.8 wt% sulfamic acid solution were added to the aforementioned solution. The absorption spectrum was tested using an UV-vis spectrophotometer and the absorption intensities at wavelengths of 220 and 275 nm were recorded. The final absorbance value was calculated using the equation: $A = A_{220nm} - 2A_{275nm}$. The concentration–absorbance curve was made using a series of standard potassium nitrate solutions.

Detection of N_2H_4

Specifically, 4 g of p-dimethylaminobenzaldehyde was dissolved into 200 mL of 95% hydrous ethanol and 20 mL of 0.6 M HCl under vigorous stirring to prepare p-dimethylaminobenzaldehyde solution with a concentration of 18.2 g L^{-1}. Then, 200 μL of the filtered and diluted reaction solution was mixed with 9.8 mL of HCl (1 M) followed by the addition of 3 mL of p-dimethylaminobenzaldehyde solution. The absorbance of the resultant solution was measured at 460 nm by UV-2600 instrument after reaction for 20 min.

Photochemical Test

Photoelectrochemical measurements were performed on a Chenhua CHI 760E electrochemical workstation by a standard three-electrode cell system in 0.1 M Na_2SO_4 electrolyte. The Pt wire, Ag/AgCl, and photocatalyst modified indium-doped tin oxide (ITO) were used as counter, reference and working electrodes, respectively. The working electrode was prepared as follows: 10 mg of catalysts was first mixed with 40 μL of 10% Nafion solution to make a slurry. Afterward, the slurry was pipetted onto a piece of ITO glass (effective area: 1 cm^2) and then dried at 60 ℃ overnight. The photocurrent was recorded under simulated sunlight using a 300 W xenon lamp (PLS-SXE300D/300DUV, Perfect Light) as light source. Electrochemical impedance spectroscopy (EIS) curves were obtained in a frequency range from 0.01 Hz to 1000 kHz under irradiation.

DFT calculations

The spin-polarization density functional theory (DFT) calculations were performed by the first-principles[5,6] within the generalized gradient approximation (GGA) using the Perdew-Burke-Ernzerhof (PBE) formulation [7]. The projected augmented wave (PAW) potentials[8,9] were chosen to describe the ionic cores and valence electrons were taken into

account using a plane wave basis set with a kinetic energy cutoff of 520 eV. Partial occupancies of the Kohn−Sham orbitals were allowed using the Gaussian smearing method and a width of 0.05 eV. The electronic energy was considered self-consistent when the energy change was smaller than 10^{-6} eV. A geometry optimization was considered convergent when the energy change was smaller than 0.05 eV $Å^{-1}$. The vacuum spacing in a direction perpendicular to the plane of the structure is 16 Å. The Brillouin zone integration is performed using 2×2×1 Monkhorst-Pack k-point sampling for a structure. In calculation of the latter two quantities, the atomic positions were fixed as in the system. The charge density (ρ) difference (Δρ) of system was calculated by the equation $\Delta\rho = \rho_{total} - \rho_A - \rho_B$, where ρ_{total} is the charge density of adsorption systems, ρ_A and ρ_B is the CoS_x and ZnS charge density, respectively. Finally, the adsorption energies (E_{ads}) of N_2 on ZnS and P-CoS_x/ZnS as well as E_{ads} of H_2O on CoS_x and P-CoS_x/ZnS were calculated as $E_{ads} = E_{ad/sub} - E_{ad} - E_{sub}$, where $E_{ad/sub}$, E_{ad}, and E_{sub} are the total energies of the optimized adsorbate/substrate system, the adsorbate in the structure, and the clean substrate, respectively. The free energy was calculated using the equation: $G = E + ZPE - TS$, where G, E, ZPE and TS are the free energy, total energy from DFT calculations, zero point energy and entropic contributions, respectively. In our calculation, the top two layers were relaxed, and the other layers fixed in surfaces structures.

Results and Discussion

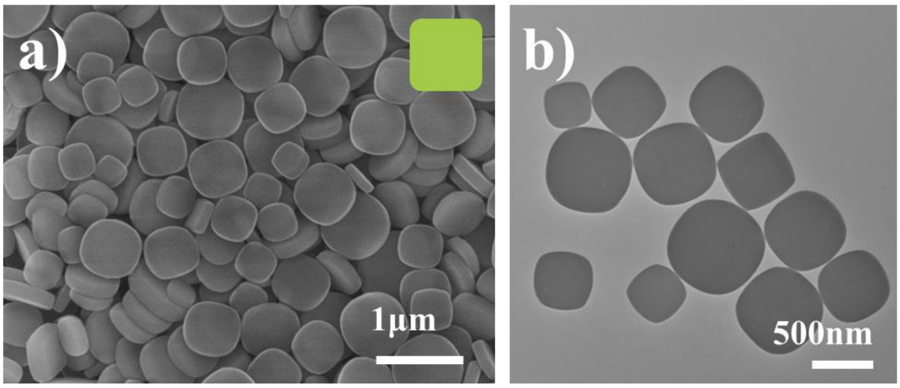

Figure S1. a) SEM and b) TEM images of NH_2-MIL-125. Scale bar: 1μm a) and 500 nm b).

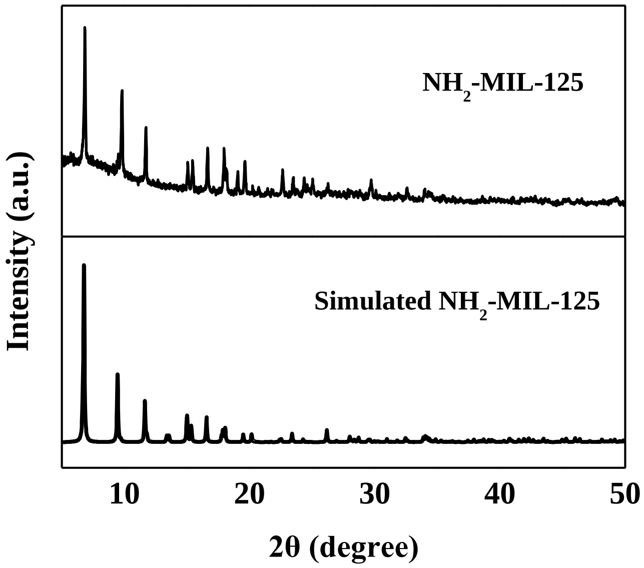

Figure S2. XRD pattern of NH_2-MIL-125.

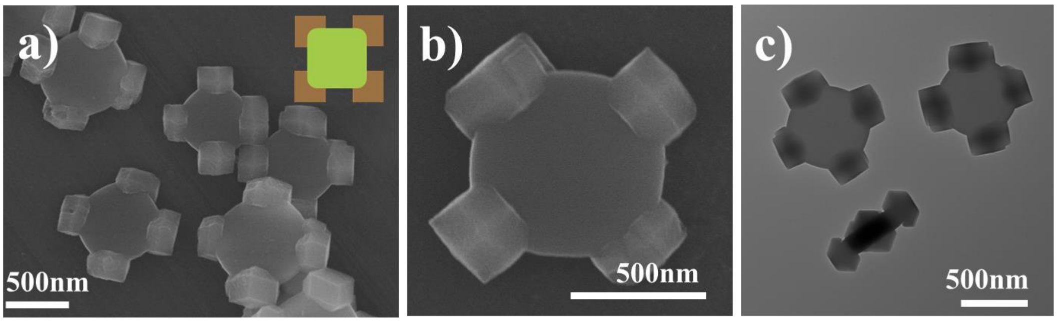

Figure S3. a-b) SEM and c) TEM images of NH_2-MIL-125@ZIF-8. Scale bar: 500 nm a-c).

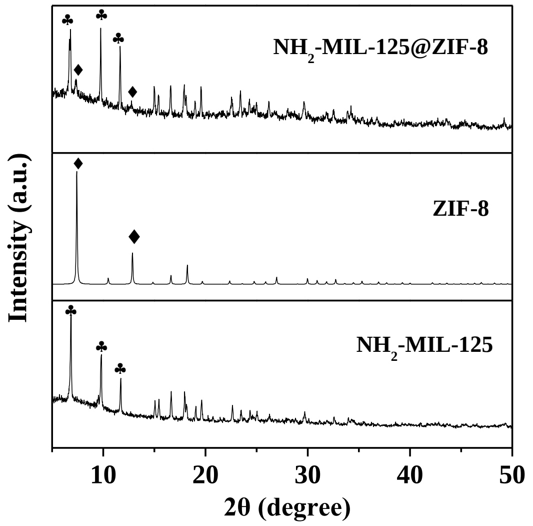

Figure S4. XRD patterns of NH_2-MIL-125@ZIF-8, ZIF-8 and NH_2-MIL-125.

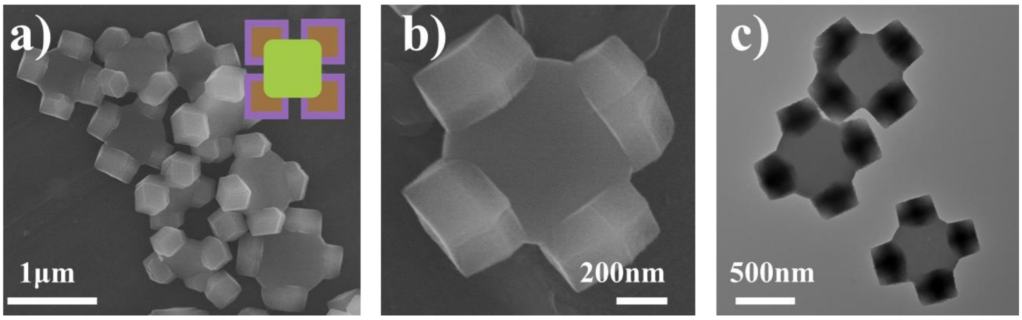

Figure S5. a-b) SEM and c) TEM images of NH_2-MIL-125@ZIF-8@ZIF-67. Scale bar: 1μm a), 200 nm b) and 500 nm c).

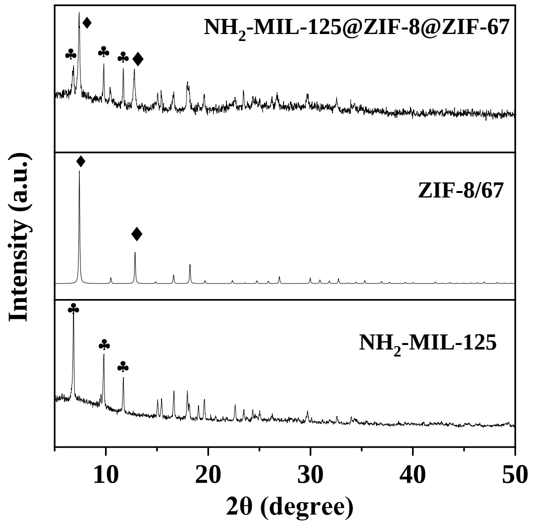

Figure S6. XRD pattern of NH_2-MIL-125@ZIF-8@ZIF-67, simulated XRD pattens of ZIF-8/67 and NH_2-MIL-125.

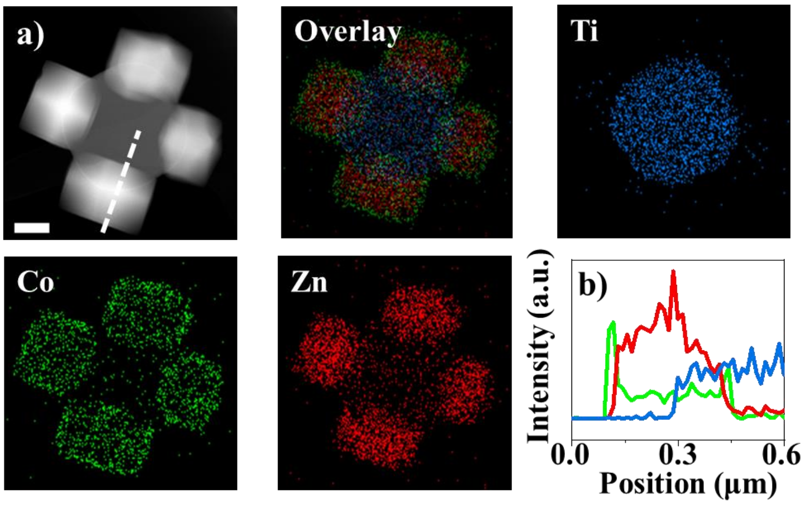

Figure S7. a) STEM and element mapping images and b) line scanning spectra of NH_2-MIL-125@ZIF-67@ZIF-8.

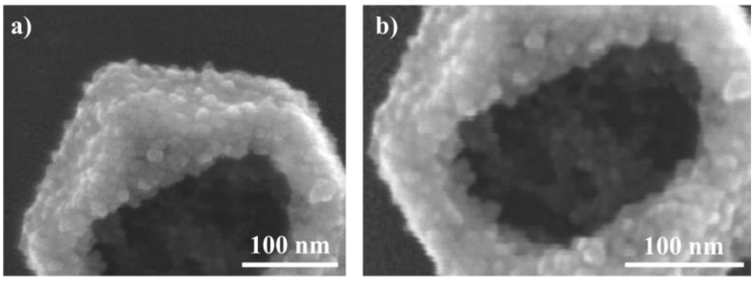

Figure S8. SEM images with higher magnifications of P-CoS_x/ZnS.

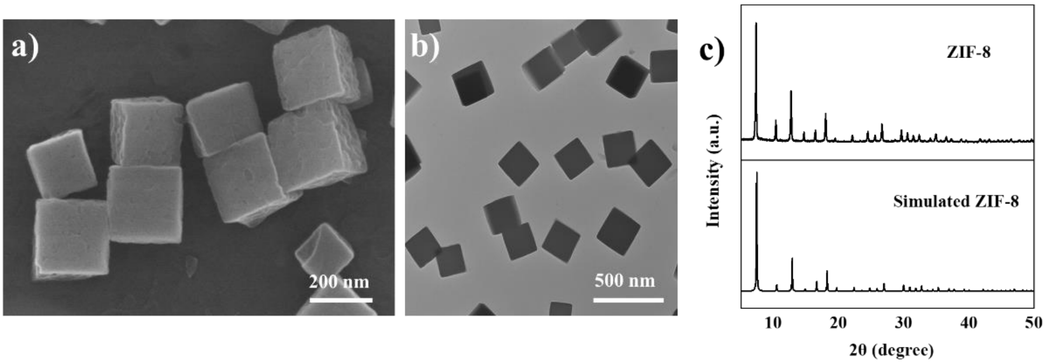

Figure S9. a) SEM, b) TEM images and c) XRD pattern of ZIF-8.

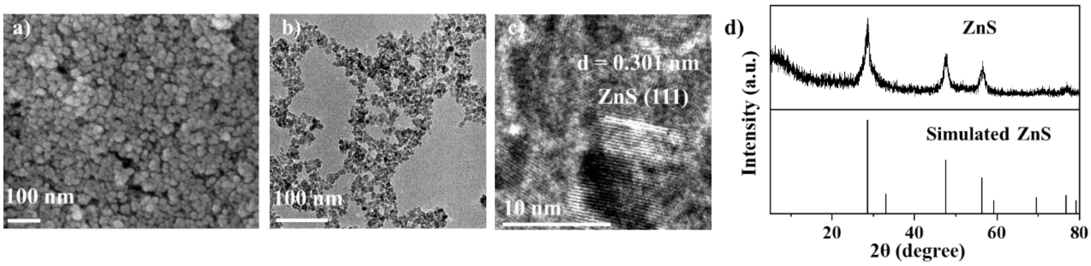

Figure S10. a) SEM, b) TEM, c) HRTEM images and d) XRD pattern of ZnS.

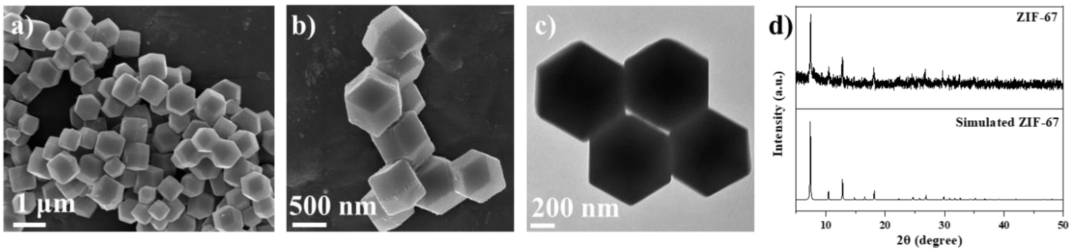

Figure S11. a-b) SEM, c) TEM images and d) XRD pattern of ZIF-67.

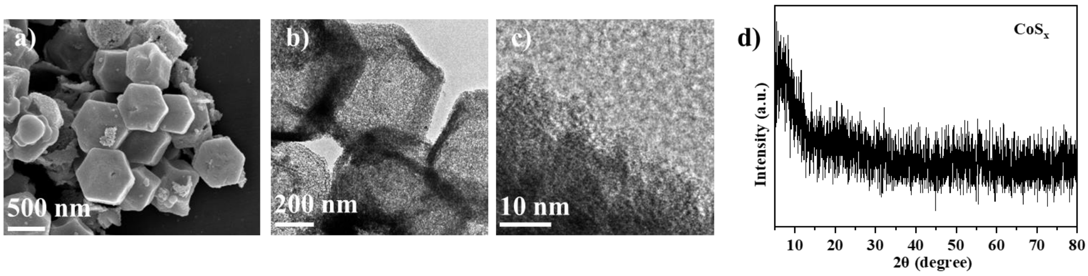

Figure S12. a) SEM, b-c) TEM images and d) XRD pattern of CoS_x.

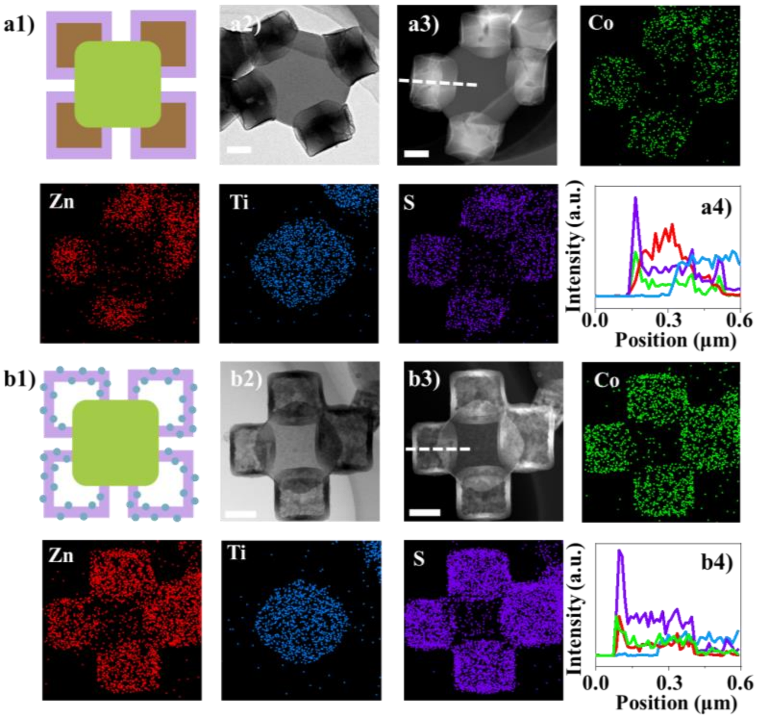

Figure S13. 1) A scheme of morphology, 2) TEM, 3) STEM and element mapping images and 4) line scanning spectra across the white line in STEM of a) NH_2-MIL-125@ZIF-67@ZIF-8-S8 and b) NH_2-MIL-125@ZIF-67@ZIF-8-S30. Scale bar: 200 nm.

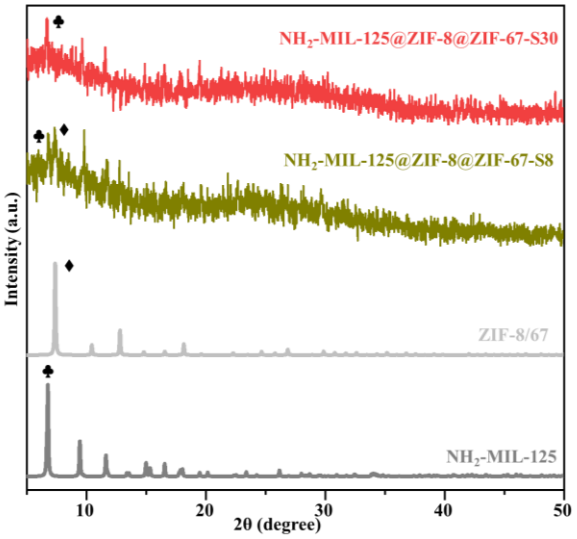

Figure S14. XRD patterns of MIL-125@ZIF-67@ZIF-8-S30, NH_2-MIL-125@ZIF-67@ZIF-8-S8, simulated ZIF-8/67 and NH_2-MIL-125.

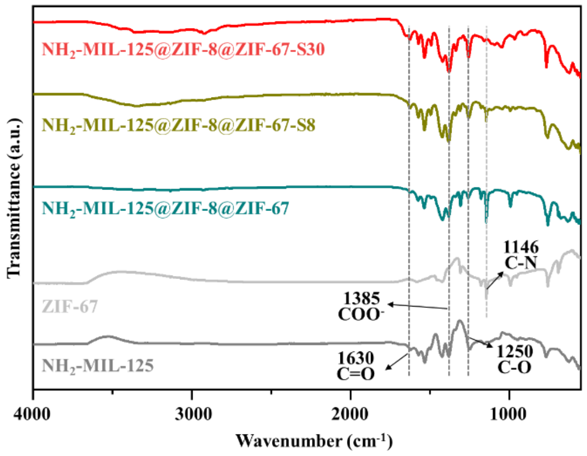

Figure S15. FTIR spectra of MIL-125@ZIF-67@ZIF-8-S30, NH_2-MIL-125@ZIF-67@ZIF-8-S8, MIL-125@ZIF-67@ZIF-8, ZIF-67 and NH_2-MIL-125.

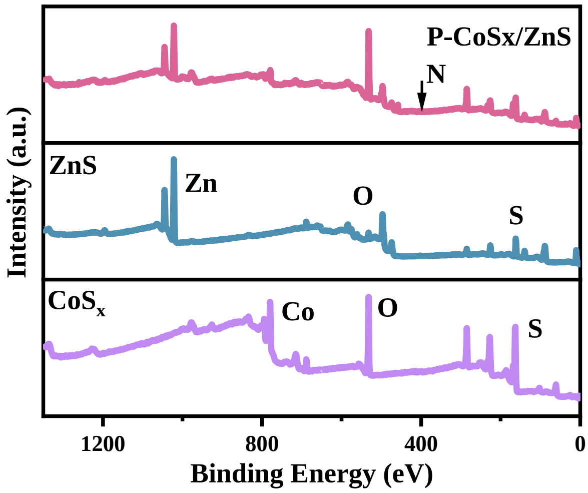

Figure S16. XPS survey spectrum of CoS_x, ZnS and P-CoS_x/ZnS.

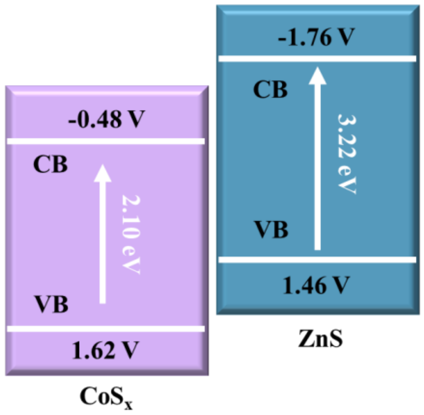

Figure S17. The electronic band structure of P-CoSx/ZnS.

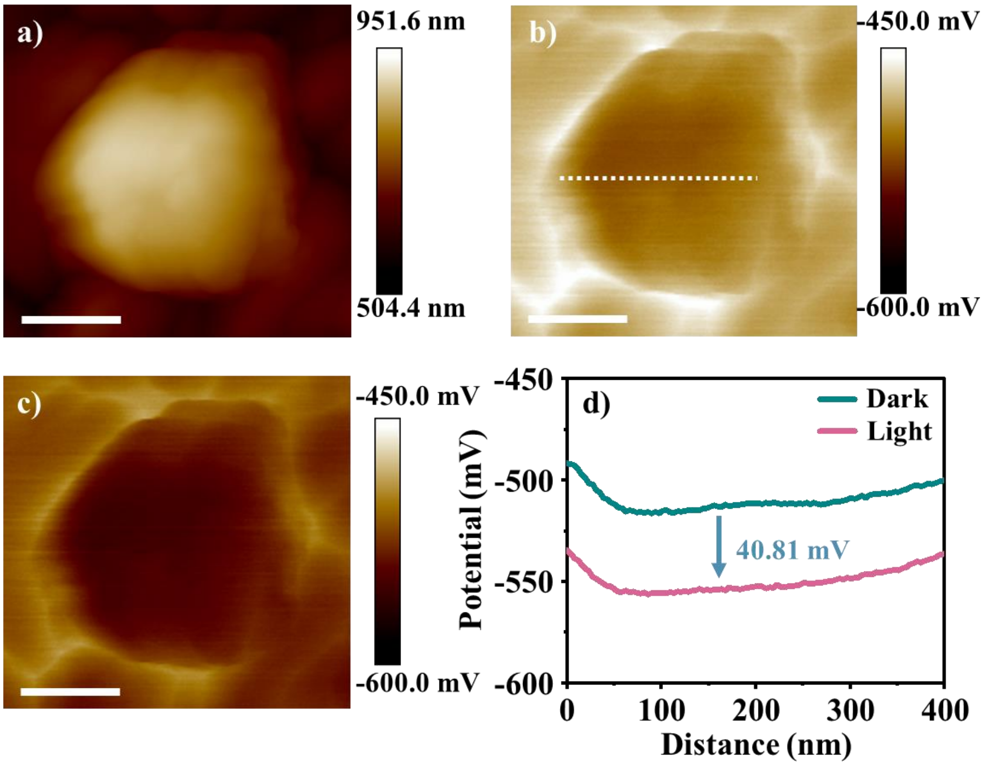

Figure S18. a) Atomic force microscopy image of P-CoS_x/ZnS. Corresponding KPFM images b) in darkness and c) under illumination. Scale bar is 200 nm. d) Line scanning surface potential along the white dashed line.

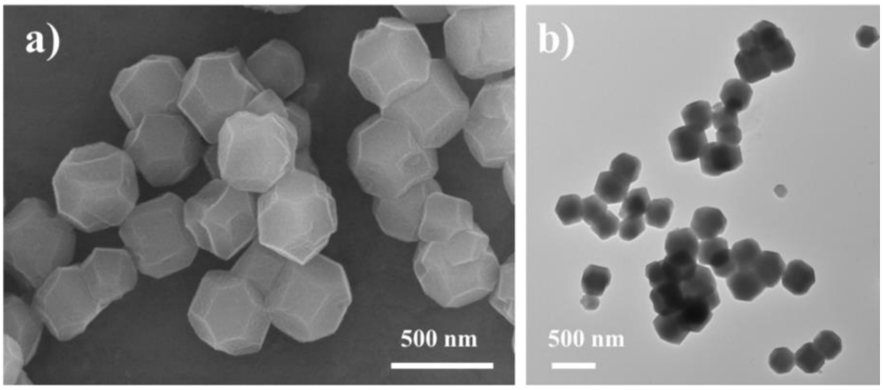

Figure S19. a) SEM and b) TEM images of ZIF-8@ZIF-67.

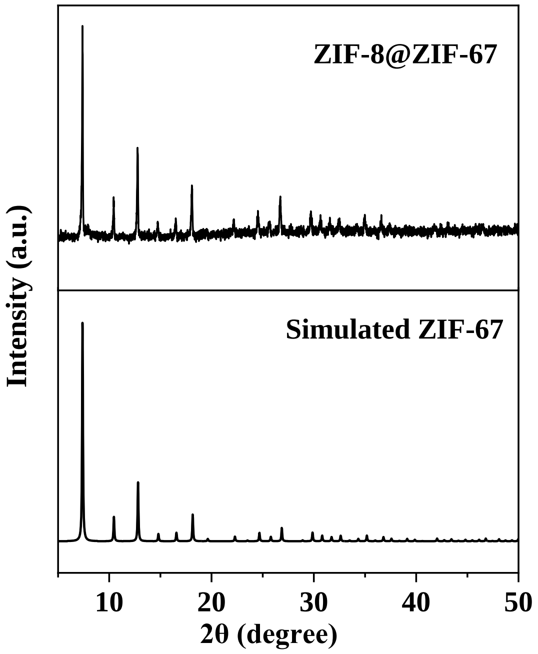

Figure S20. XRD pattern of ZIF-8@ZIF-67.

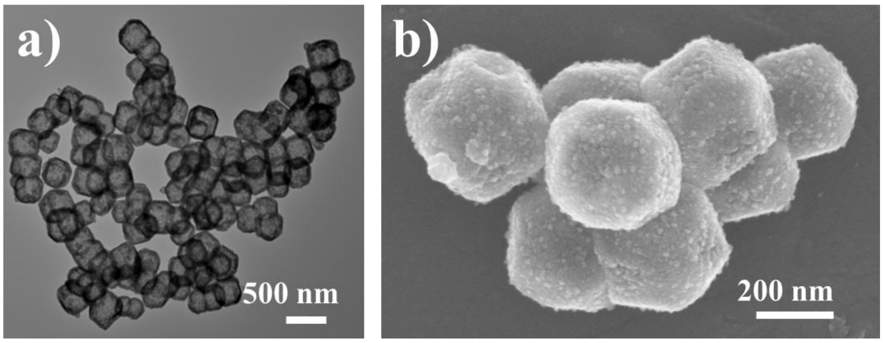

Figure S21. a) TEM and b) SEM images of H-CoS_x/ZnS.

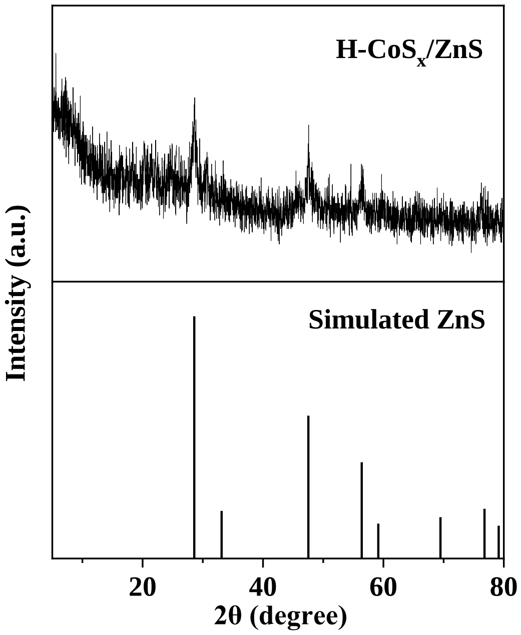

Figure S22. XRD pattern of H-CoS_x/ZnS.

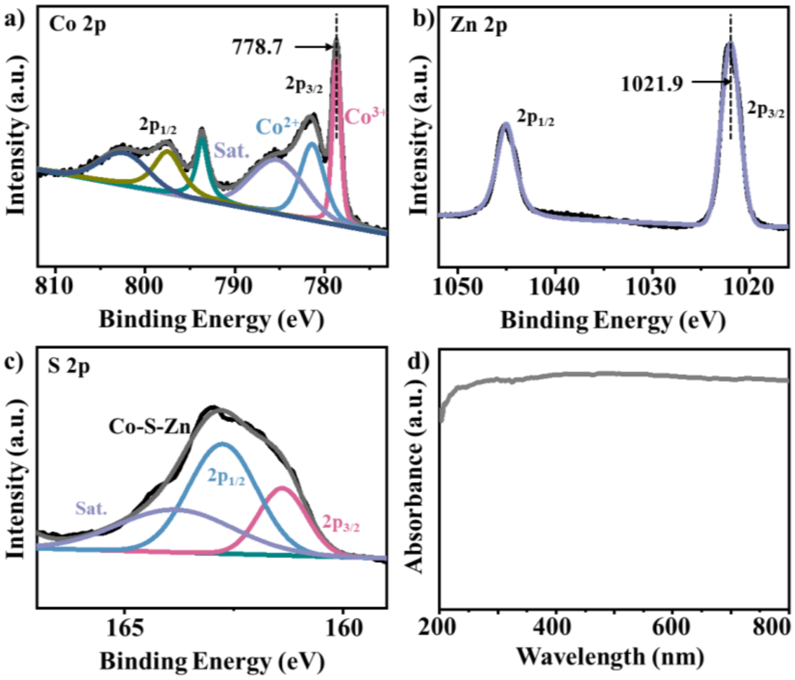

Figure S23. High resolution XPS spectra of a) Co 2p, b) Zn 2p and c) S 2p. d) UV–vis DRS of H-CoS_x/ZnS.

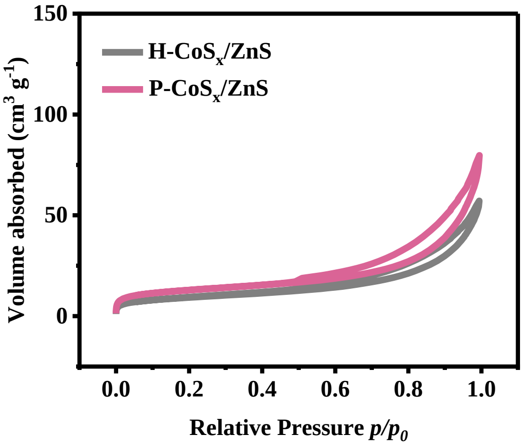

Figure S24. Nitrogen adsorption-desorption isotherms of P-CoS_x/ZnS and H-CoS_x/ZnS.

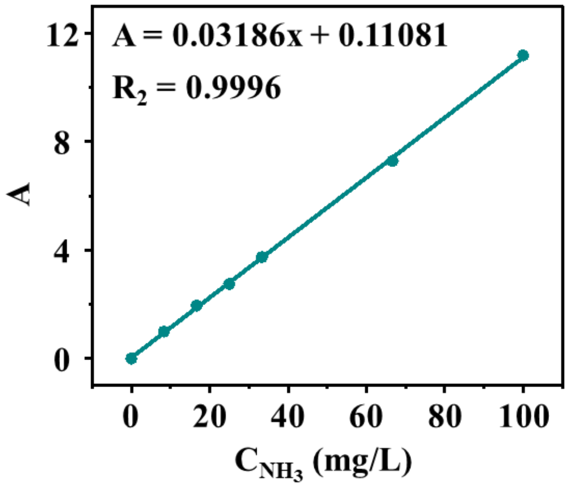

Figure S25. Standard curve for NH_3 quantification.

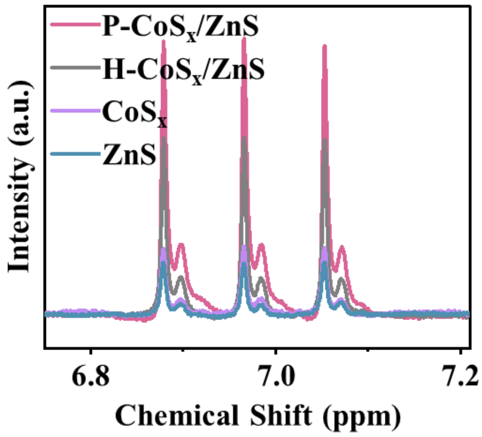

Figure S26. ^{1}H NMR spectra of the reaction solution of different samples.

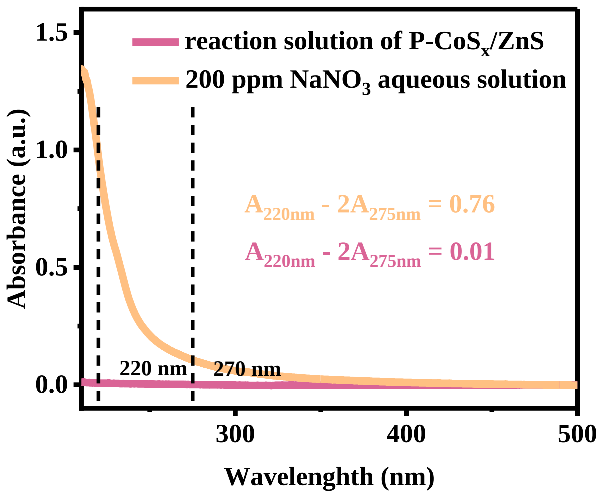

Figure S27. UV-vis spectra of NO_3^- detection.

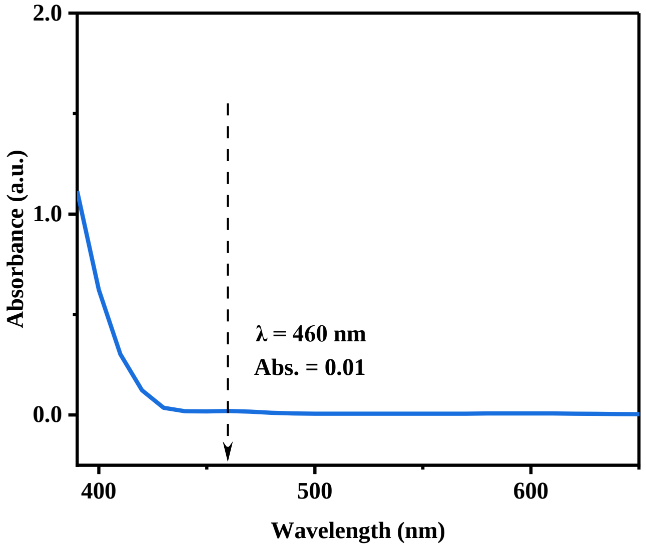

Figure S28. UV-vis spectra of N_2H_4 detection.

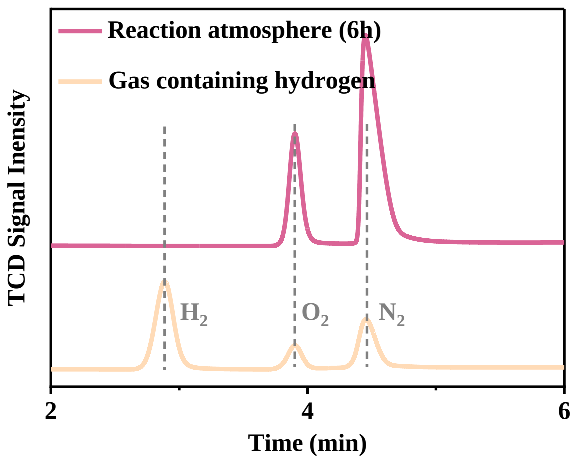

Figure S29. Gas chromatograph spectra of reaction atmosphere.

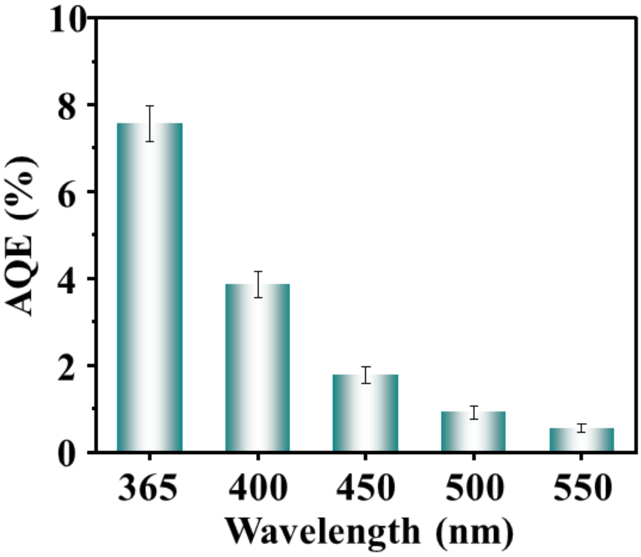

Figure S30. AQEs of photocatalytic N_2 fixation on P-CoS_x/ZnS at different wavelengths.

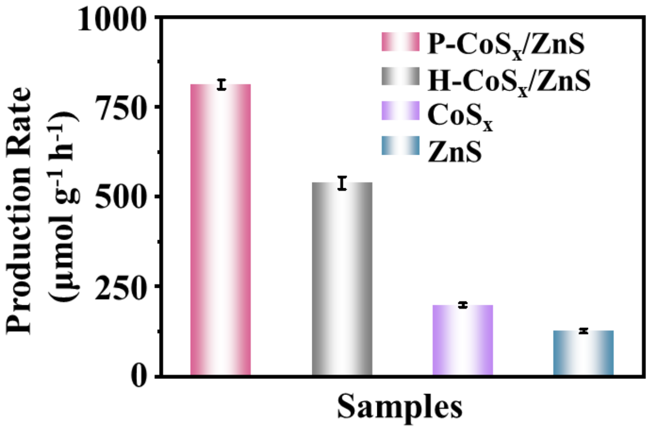

Figure S31. O_2 production rate of P-CoS_x/ZnS, H-CoS_x/ZnS, CoS_x and ZnS.

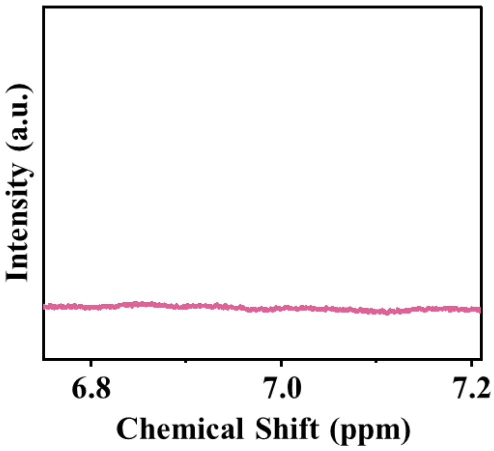

Figure S32. The ^{1}H NMR spectra of the reaction solution of P-CoS_x/ZnS using Ar as feeding gas.

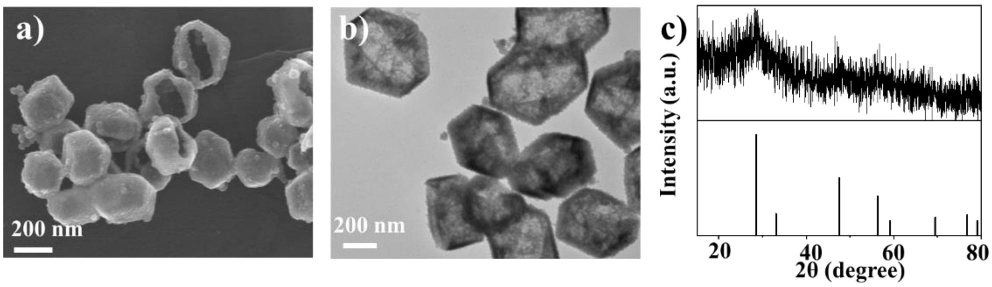

Figure S33. a) SEM, b) TEM images and c) XRD pattern of P-CoS_x/Zn after five cycles.

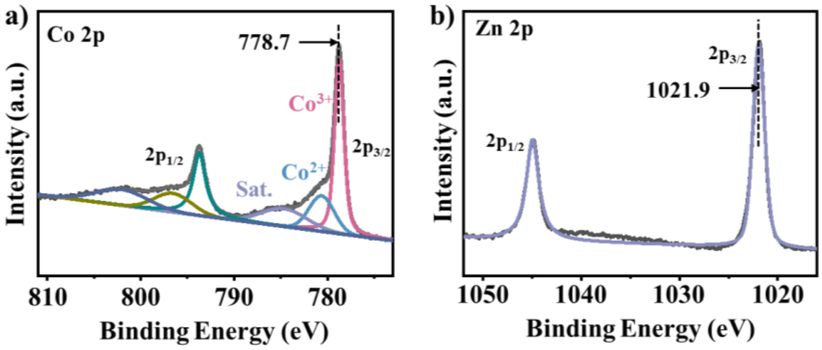

Figure S34. High resolution XPS spectra of a) Co 2p and b) Zn 2p of P-CoS_x/ZnS after five cycles.

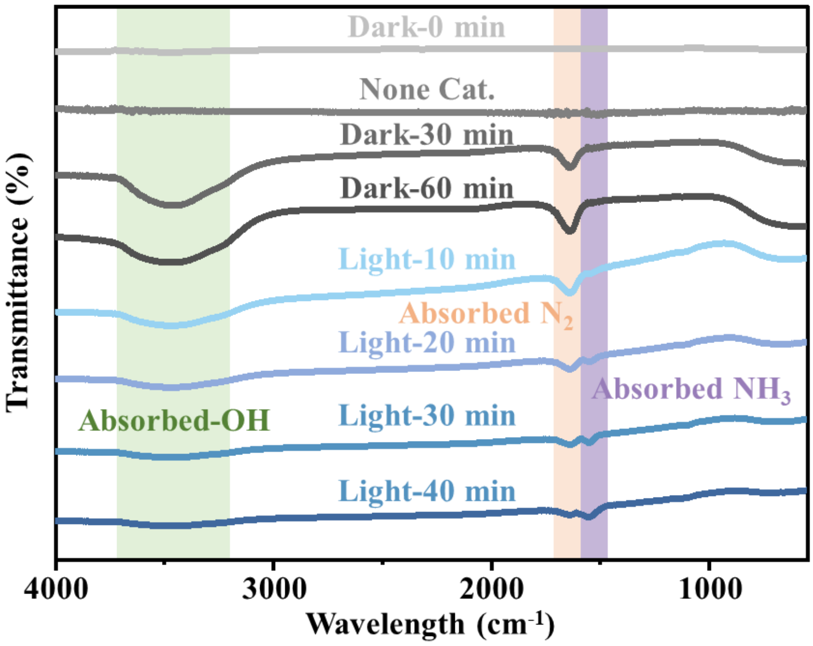

Figure S35. In situ DRIFT spectra of P-CoS_x/ZnS during the photocatalytic N_2 fixation.

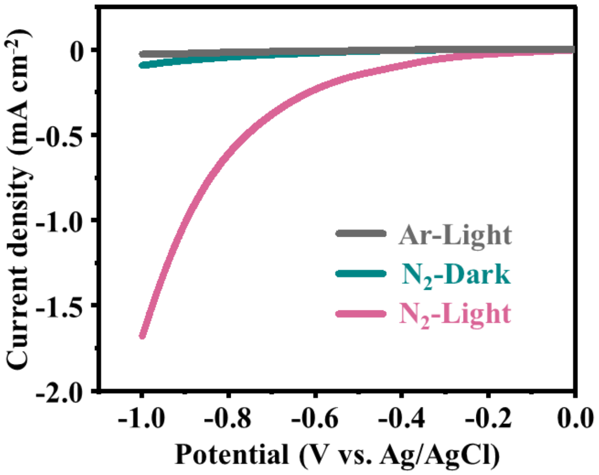

Figure S36. LSV curves of P-CoS_x/ZnS under different conditions.

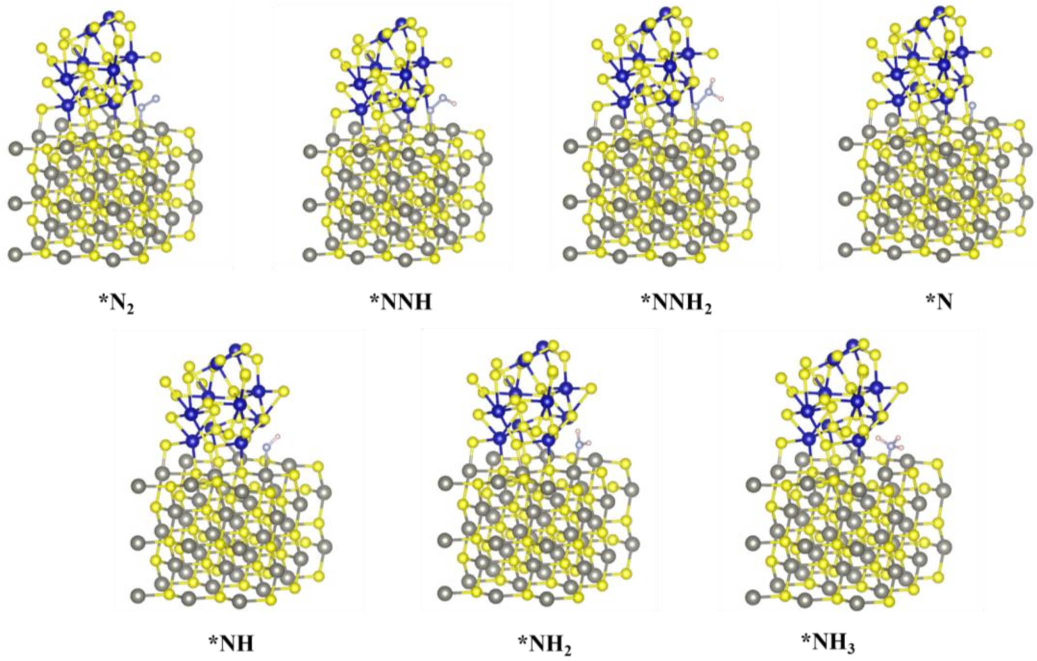

Figure S37. The adsorption diagrams of the N_2 fixation process over P-CoS_x/ZnS

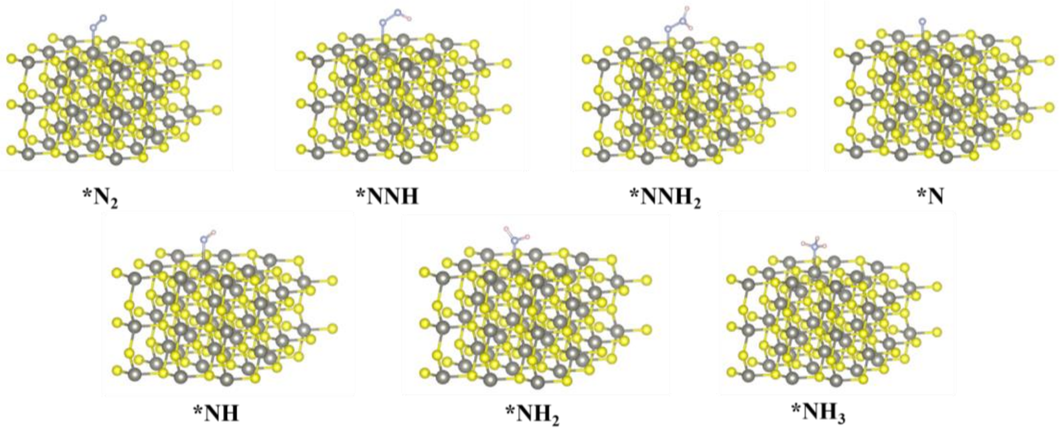

Figure S38. The adsorption diagrams of the N_2 fixation process over of ZnS.

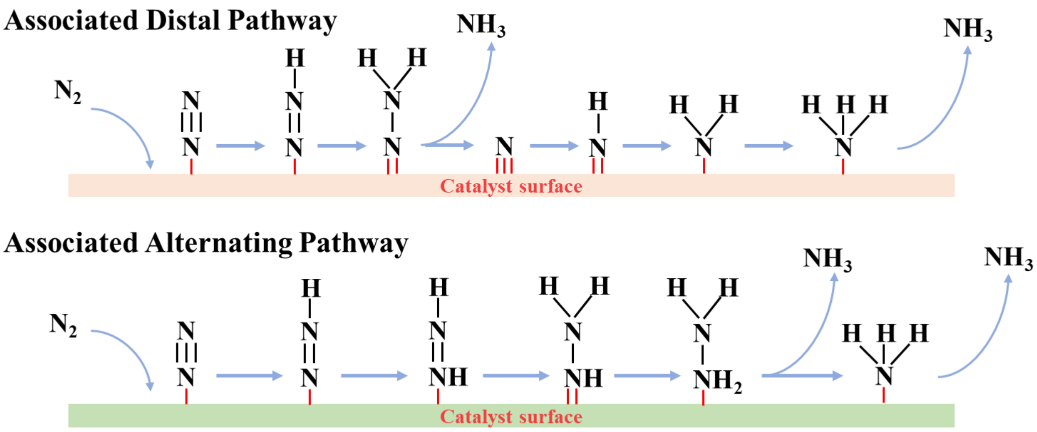

Figure S39. The distal and alternating pathways for photocatalytic N_2 fixation.

Table S1. BET specific surface areas and pore volumes of P-CoS_x/ZnS and H-CoS_x/ZnS.

Sample	BET specific surface area m^2 g^{-1}	Pore volume (cm^3 g^{-1})
P-CoS_x/ZnS	42.4	0.119
H-CoS_x/ZnS	34.1	0.086

Table S2. The AQE for photocatalytic N_2 fixation in recent reports

Catalyst	AQE	Reference
P-CoS_x/ZnS	7.56% at 365 nm	This work
Gd-IHEP-8	1.72% at 365 nm	[10]
$Ni_2P/Cd_{0.5}Zn_{0.5}S$	4.32% at 420 nm	[11]

Ru-Vs-CS-CN	1.28% at 400 nm	[12]
Bi_5O_7Br	2.3% at 420 nm	[13]
CuCr-LDH	0.44% at 380 nm	[14]
Defect TiO_2	0.7 % ≤ 350 nm	[15]
$C_3N_4/MoS_2/Mn_3O_4$	1.2% at 420 nm	[16]
BMO-Br-Ov	0.52% at 420 nm	[17]
Au@MOF	1.54% at 520 nm	[18]
g-C_3N_4-N_{3C}-0.3	7.79% at 370 nm	[19]

Table S3. The onset potential at 0.1 mA cm^{-2} and current density at -1 V (vs. Ag/AgCl) of P-CoS_x/ZnS, CoS_x and ZnS.

Sample	Onset potential (V vs. AgCl)	Current density (mA cm^{-2})
P-CoSx/ZnS	-0.41	1.68
CoSx	-0.49	0.43
ZnS	-0.71	0.17

References

1. Dan-Hardi M, Serre C, Frot T *et al.* A New Photoactive Crystalline Highly Porous Titanium(IV) Dicarboxylate. *J Am Chem Soc*. 2009; **131**: 10857.

2. Lu X, Quan L, Hou H *et al.* Fabrication of 1D/2D Y-doped $CeO_2/ZnIn_2S_4$ S-scheme photocatalyst for enhanced photocatalytic H_2 evolution. *J Alloy Compd*. 2022; **925**: 166552.

3. Zhao Y, Shi R, Bian X *et al.* Ammonia Detection Methods in Photocatalytic and Electrocatalytic Experiments: How to Improve the Reliability of NH_3 Production Rates? *Adv Sci*. 2019; **6**: 1802109.

4. Hirakawa H, Hashimoto M, Shiraishi Y *et al.* Photocatalytic Conversion of Nitrogen to Ammonia with Water on Surface Oxygen Vacancies of Titanium Dioxide. *J Am Chem Soc*. 2017; **139**: 10929.

5. Kresse G and Furthmuller J. Efficiency of ab-initio total energy calculations for metals and semiconductors using a plane-wave basis set. *Comp Mater Sci*. 1996; **6**: 15.

6. Kresse G and Furthmuller J. Efficient iterative schemes for ab initio total-energy calculations using a plane-wave basis set. *Phys Rev B* 1996; **54**: 11169.

7. Perdew JP, Burke K and Ernzerhof M. Generalized Gradient Approximation Made Simple. *Phys Rev Lett*. 1996; **77**: 3685.

8. Blöchl PE. Projector augmented-wave method. *Phys Rev B*. 1994; **50**: 17953.

9. Kresse G and Joubert D. From Ultrasoft Pseudopotentials to the Projector Augmented-Wave Method. *Phys Rev B*. 1999; **59**: 1758.

10. Hu KQ, Qiu PX, Zeng LW *et al.* Solar-Driven Nitrogen Fixation Catalyzed by Stable Radical-Containing MOFs: Improved Efficiency Induced by a Structural Transformation. *Angew Chem Int Ed*. 2020; **59**: 20666.

11. Ye L, Han C, Ma Z *et al.* Ni_2P loading on $Cd_{0.5}Zn_{0.5}S$ solid solution for exceptional photocatalytic nitrogen fixation under visible light. *Chem Eng J*. 2017; **307**: 311.

12. Yuan J, Yi X, Tang Y *et al.* Efficient Photocatalytic Nitrogen Fixation: Enhanced Polarization, Activation, and Cleavage by Asymmetrical Electron Donation to N-N Bond. *Adv Funct Mater*. 2019; **30**: 1906983.

13. Wang S, Hai X, Ding X *et al.* Light-Switchable Oxygen Vacancies in Ultrafine Bi_5O_7Br Nanotubes for Boosting Solar-Driven Nitrogen Fixation in Pure Water. *Adv Mater*. 2017; **29**: 1701774.

14. Zhao Y, Zhao Y, Waterhouse GIN *et al.* Layered-Double-Hydroxide Nanosheets as Efficient Visible-Light-Driven Photocatalysts for Dinitrogen Fixation. *Adv Mater*. 2017; **29**: 1703828.

15. Zhao Y, Zhao Y, Shi R *et al.* Tuning Oxygen Vacancies in Ultrathin TiO_2 Nanosheets to Boost Photocatalytic Nitrogen Fixation up to 700 nm. *Adv Mater*. 2019; **31**: 1806482.

16. Li H, Liu Y, Liu Y *et al.* Efficient Visible Light Driven Ammonia Synthesis on Sandwich Structured $C_3N_4/MoS_2/Mn_3O_4$ catalyst. *Appl Catal B: Environ*. 2021; **281**: 119476.

17. Wang G, Huo T, Deng Q *et al.* Surface-layer bromine doping enhanced generation of surface oxygen vacancies in bismuth molybdate for efficient photocatalytic nitrogen fixation. *Appl Catal B: Environ*. 2022; **310**: 121319.

18. Chen L-W, Hao Y-C, Guo Y *et al.* Metal–Organic Framework Membranes Encapsulating Gold Nanoparticles for Direct Plasmonic Photocatalytic Nitrogen Fixation. *J Am Chem Soc*. 2021; **143**:

5727.

19. Xue Y, Ma C, Yang Q *et al.* Construction of g-C_3N_4 with three coordinated nitrogen (N_3C) vacancies for excellent photocatalytic activities of N_2 fixation and H_2O_2 production. *Chem Eng J*. 2023; **457**: 141146.
